# Design and Analysis of Super Wideband Antenna for Microwave Applications

**DOI:** 10.3390/s21020477

**Published:** 2021-01-12

**Authors:** Warsha Balani, Mrinal Sarvagya, Ajit Samasgikar, Tanweer Ali, Pradeep Kumar

**Affiliations:** 1 School of ECE, REVA University, Bangalore 560064, India; balani.warsha@gmail.com (W.B.); mrinalsarvagya@gmail.com (M.S.); 2 MMRFIC Technology Private Limited, Bangalore 560016, India; ajitsamasgikar@gmail.com; 3 Department of Electronics & Communication, Manipal institute of Technology, Manipal Academy of Higher Education, Manipal, Karnataka 576104, India; 4 Discipline of Electrical, Electronic and Computer Engineering, University of KwaZulu-Natal, Durban 4041, South Africa

**Keywords:** bandwidth dimension ratio (BDR), fidelity factor (FF), group delay, super wideband (SWB), semi-elliptical

## Abstract

In this article, a compact concentric structured monopole patch antenna for super wideband (SWB) application is proposed and investigated. The essential characteristics of the designed antenna are: (i) to attain super-wide bandwidth characteristics, the proposed antenna is emerged from a traditional circular monopole antenna and has obtained an impedance bandwidth of 38.9:1 (ii) another important characteristic of the presented antenna is its larger bandwidth dimension ratio (BDR) value of 6596 that is accomplished by augmenting the electrical length of the patch. The electrical dimension of the proposed antenna is 0.18λ×0.16λ (λ corresponds to the lower end operating frequency). The designed antenna achieves a frequency range from 1.22 to 47.5 GHz with a fractional bandwidth of 190% and exhibiting S_11_ < −10 dB in simulation. For validating the simulated outcomes, the antenna model is fabricated and measured. Good conformity is established between measured and simulated results. Measured frequency ranges from 1.25 to 40 GHz with a fractional bandwidth of 188%, BDR of 6523 and S_11_ < −10 dB. Even though the presented antenna operates properly over the frequency range from 1.22 to 47.5 GHz, the results of the experiment are measured till 40 GHz because of the high-frequency constraint of the existing Vector Network Analyzer (VNA). The designed SWB antenna has the benefit of good gain, concise dimension, and wide bandwidth above the formerly reported antenna structures. Simulated gain varies from 0.5 to 10.3 dBi and measured gain varies from 0.2 to 9.7 dBi. Frequency domain, as well as time-domain characterization, has been realized to guide the relevance of the proposed antenna in SWB wireless applications. Furthermore, an equivalent circuit model of the proposed antenna is developed, and the response of the circuit is obtained. The presented antenna can be employed in L, S, C, X, Ka, K, Ku, and Q band wireless communication systems.

## 1. Introduction

With the present development and expeditious growth in the area of wireless communication, the design of antennas for portable devices with enhanced radiation characteristics is receiving utmost importance. Compactness, high data rate, efficiency, capacity, etc., are the major considerable criteria for the next generation antennas. The frequency spectrum assigned for ultra-wideband (UWB) applications by the Federal Communication Commission (FCC) in 2002 from 3.1 to 10.6 GHz extends only for short-range communication [1]. Thus, to meet the interest for both long and short distance communication, researchers have initiated efforts to embed super wideband (SWB) technology into wireless communication devices. SWB antenna should be efficient in operating over a UWB bandwidth as assigned by the FCC and also should cover up cellular and radar communication frequency bands. Numerous research works are carried out in the design of SWB antennas with respect to higher bandwidth dimension ratio (BDR) and lesser electrical dimensions. Antenna that can contribute to minimal levels on a decade bandwidth (10:1) can be called SWB antenna. SWB technology provides larger bandwidth and high data rate which can also be utilized to broadcast the voice, video and data at a faster rate. This technology at present is being employed in ranging and monitoring systems, for both civil and military applications.

The major challenge faced by the researcher is to design planar antenna with large impedance bandwidth, larger value of BDR and stabilized radiation properties with less distortion mainly at higher frequency. Therefore, considering these demands, many SWB antennas have been designed and presented in the literature.

To embed the benefits of SWB technology in practical wireless applications, multidimensional research has been undertaken for diverse antenna configurations. For the purpose of achieving wider bandwidth, many different kinds of bandwidth enhancement techniques, such as modification in the ground plane and the patch [2,3,4,5,6,7,8] along with a diverse range of feeding techniques, such as microstrip tapered feedline [9,10,11,12,13] and Coplanar Waveguide (CPW) feed [14,15,16,17,18,19,20,21], have been proposed by antenna designers. To miniaturize the dimension of antenna without impacting the impedance bandwidth, self-similarity structures, such as fractal geometry [22,23,24,25,26,27], have been envisioned in the literature.

Rahman et al. [2] proposed a crescent-shaped radiating patch antenna with a rectangular slotted partial ground to cover frequency spectrum from 2.5 to 29.0 GHz. However, uneven gain is observed over the entire range of frequency. Lingsheng Yang et al. [3] proposed an SWB antenna fed by a coplanar waveguide. Ground plane is a symmetric smoothing structure to improve the impedance bandwidth from 0.4 to 20 GHz. Similarly, SWB patch antenna with frequency selective structure is presented for frequency bands from 3 to 20 GHz [4]. However, the major problem associated with the aforementioned structures is, in spite of having wide impedance bandwidth, the designed antenna structures are not appropriate for low frequency band applications, such as Bluetooth, GPS and GSM. Ramanujam [5] proposed an SWB antenna with hybrid structure fed by an stepped microstrip line. Two semi-circular parasitic patches on the radiator enhances the impedance bandwidth from 24 to 40 GHz. However, unstable radiation patterns are observed over the entire frequency band. Circular-shaped patch antenna fed by a microstrip feed line has been presented to operate at a range of 2.4–28.4 GHz [6]. However, negative gains are observed at lower frequencies. An octagonal ring-shaped antenna is proposed by Okan. A stub at the top right corner of the patch is used to enhance the impedance bandwidth from 2.59 to 31.14 GHz [7]. Polygonal-shaped patch radiator with circular slotted ground plane, which covers the frequency range of 2.25–11.05 GHz, has been presented by Syeed et al. [8]. However, characteristics of the antenna with regard to time domain performance need to be evaluated.

Elliptical-shaped patch, fed with tapered microstrip feedline, is designed to exhibit extremely broad bandwidth from 640 MHz to 16 GHz. Although a higher peak gain is stated, the structure of the antenna is large 150 mm × 150 mm to be incorporated into portable devices [9]. A Hanning function-based tapered microstrip monopole antenna is presented to provide a wide frequency range from 2.5 to 110 GHz [10]. However, impedance matching is fairly good for the overall frequency range. Awan [11] proposed an SWB slotted Y-shaped antenna with impedance bandwidth from 15.4 to 71.53 GHz. However, the fabricated antenna performance needs to be evaluated. Similarly, a semicircular-shaped patch with tapered feedline and trapezoid ground plane is proposed by Samsuzzaman et al. [12] to cover frequency spectrums from 1.30 to 20 GHz. However, phase variation across the overall frequency band of operation is not linear, leading to pulse distortion. A partial circular monopole antenna with elliptical slot, which enhances the lower operating frequency, which in turn increases the bandwidth dimension ratio to 6975.22, is proposed [13]. In order to augment the impedance bandwidth and to reduce the coupling effect, a tapered microstrip line feed with notch loaded elliptical ground plane is suggested. The proposed antenna achieves a frequency range of 0.96 GHz to 10.9 GHz with a bandwidth ratio of 11.35:1. However, the impedance bandwidth of the proposed antenna is less and a very low gain of 0.5 dBi is observed at the lowest operating frequency.

A CPW-fed propeller-structured monopole antenna for SWB application is demonstrated to provide a good impedance bandwidth from 3 to 35 GHz; however, it suffers from a lower BDR value (809) due to its large electrical dimensions [14]. In [15], a CPW-fed antenna with inverted triangular shaped patch is designed to exploit across the frequency range from 3.06 to 35 GHz. However, the gain of the proposed antenna structures has not been reported. Singhal et al. [16] presented a CPW-fed hexagonal-shaped Sierpinski fractal antenna loaded with two iteration of square slot to attain wide bandwidth from 3.4 to 37.4 GHz. Similarly, a novel compact semi-circular triangular antenna with CPW feed for SWB communication applications has been proposed to attain a good impedance bandwidth from 4.9 to 25 GHz [17]. However, the designed antenna could not support the lower frequency range of applications such as L and S band. A staircase approach on the rectangular patch along with CPW feed is adopted to achieve wide bandwidth of 3.15–32 GHz. However, negative gains are observed throughout the bandwidth [18]. In order to achieve wide bandwidth of 3.5–37.2 GHz, a π-shaped radiator was reported by Singhal et al. [19]. However, improper impedance bandwidth matching is observed at higher frequency. Likewise, a CPW-fed asymmetric ground plane fractal SWB antenna is proposed to achieve an operating frequency range from 2.75 to 71 GHz [20]. However, impedance matching is not so good over the band of operation. In [21], the author presented a co-planner waveguide-fed square monopole antenna which is modified by adding two stubs at the opposite side, which in turn increases the electrical length of monopole resulting in large BDR 7871.49. The designed antenna operates at a frequency range of 0.95–13.8 GHz, with a radiation efficiency of 80% and gain of 5.8 dBi at 13.8 GHz. Radiation efficiency is slightly decreasing as frequency increases which is due to the lossy nature of Rogers RT/Duroid 5880 substrate material and also due to the presence of losses in the surface wave.

A rectangular patch with Giusepe Peano fractal geometry is designed to operate over a wide frequency range from 3 to 26 GHz [22]. However, the aforesaid designed antenna does not cover lower frequency range of operation. A CPW-fed octagonal-shaped fractal radiator with defective ground structure has been presented by Singhal to acquire impedance bandwidth of 3.8–68 GHz [23]. However, distorted radiation pattern is observed at frequency greater than 10 GHz. A circular metallic patch nested with three iterations of Apollonius circles is designed to function over the bandwidth of 3–60 GHz [24]. Similarly, a star-shaped fractal antenna is proposed to function over the overall frequency range from 17.22 to 180 GHz [25]. However, experimental validation needs to be carried out for the above proposed antenna. An elliptical ground with a triangle circular patch fractal antenna was proposed for broad impedance bandwidth of 1.4–20 GHz [26]. An octagonal-shaped patch with Koch fractal geometry has been proposed to achieve bandwidth from 2 to 30 GHz [27]. However, time domain characterization needs to be carried out for validating the performance of antenna.

### Contribution

In this article, a microstrip-fed printed monopole antenna for SWB application is investigated. The proposed antenna is derived from a conventional circular monopole antenna. To obtain wide bandwidth and matching, conventional circular monopole antenna has been modified by adjoining a pair of ears at the upper part of the radiator and also by modifying its partial ground plane. The novelty in the structure is brought by introducing a concentric Mickey-Mouse-shaped slot inside the patch which facilitates in extending the impedance bandwidth. The introduction of the slot helps to increase the current path length and thus keep the dimensions of the antenna intact. Two significant contributions of the presented paper are:

- A semi-elliptical ground plane, along with the notch, is employed to lower the effect of capacitance, which in turn is used to nullify the inductive effect of patch resulting in an improved impedance matching.
- Drawing the most attention for the proposed radiator is its large BDR and its wide band width, which is attained by augmenting the electrical length of the radiator. The proposed antenna structure achieves frequency range from 1.22–47.5 GHz (VSWR < 2) with fractional bandwidth of 190% and bandwidth ratio of 38.9:1.

Antenna parameters, which are employed in describing the antenna performance, are impedance bandwidth, radiation pattern, directivity, input impedance and gain. In this paper, frequency domain characteristics with regard to radiation pattern, reflection coefficient and gain are investigated to evaluate antenna performance. In addition, for SWB applications where a very short duration pulse of a few pico-seconds is utilized for transmission and reception, good time domain behavior is equally considered important to determine the antenna characteristics for practical applications. Therefore, time-domain characteristics are examined to evaluate the applicability of the designed antenna for pulse-based communication systems. This paper also focuses on developing an equivalent circuit model of the presented patch antenna. The circuit simulation is conducted using the ADS Software tool.

This paper is organized as follows: Antenna geometry is discussed in Section 2. All the important parameters that control the bandwidth and impedance matching are described in Section 3. Experimental results with regard to radiation pattern, reflection coefficient and gain are computed theoretically and also measured using a Vector Network Analyzer and described in Section 4. Section 5 concentrates on the modeling of the equivalent circuit for the proposed antenna. Section 6 describes the time-domain analysis. Final conclusion is presented in Section 7.

## 2. Antenna Configuration

**Figure 1:**
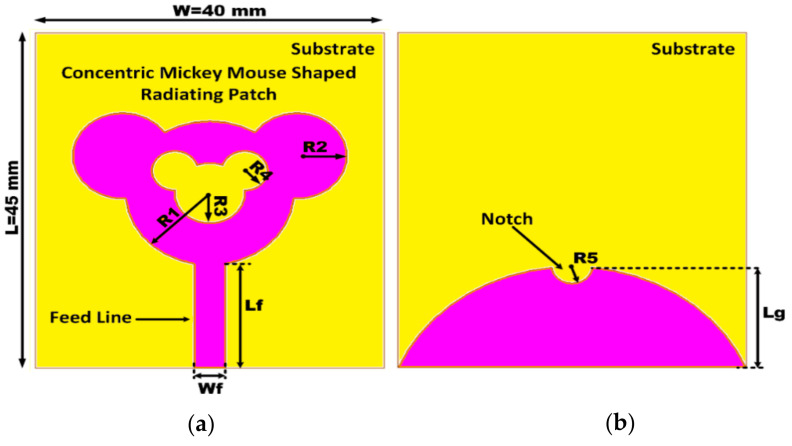
Proposed concentric-shaped super wideband (SWB) antenna; (**a**) top view, (**b**) bottom view.

The configuration of the designed antenna structure is illustrated, and a detailed dimension layout is given in Figure 1. The optimal dimensions of the designed antenna structure are given in Table 1. The substrate adopted for designing the proposed radiating structure is a Rogers RT/Duroid 5880 dielectric material with a dielectric constant of ε=2.2, loss tangent of δ=0.0009 and thickness of 1.57 mm. It can be noticed that the antenna structure is composed of a concentric Mickey-Mouse-shaped radiating patch and a notch-loaded semi-elliptical ground plane.

## 3. Design Analysis of the Proposed Antenna

This section gives the design of the proposed concentric-shaped SWB antenna as illustrated in Figure 1. Wide bandwidth is obtained by carving slots on the Mickey-Mouse-shaped radiator patch and on the semi-elliptical ground plane. The electrical dimension of the radiator is increased by the introduction of slot at the top portion of the radiator. Addition of slot disturbs the distribution of the current, thereby increasing the effective inductance and capacitance, which results in minimizing the lowest operating frequency from 2.2 to 1.2 GHz and improves the BDR of the proposed radiator. Proposed antenna analysis is carried out using Ansys High Frequency Structure Simulator (HFSS) Electromagnetic (EM) simulator.

The lower frequency of the proposed antenna is calculated by Equation (1).

(1) $$f_{L}=\frac{c}{2\times 2\cdot \frac{A}{r}\sqrt{\varepsilon_{eff}}}$$

(2) $$\varepsilon_{eff}=\frac{\varepsilon_{r}+1}{2}+\frac{\varepsilon_{r}-1}{2}{\left[1+12\frac{h}{W_{F}}\right]}^{-\frac{1}{2}}$$

where $\varepsilon_{eff}$ is the effective dielectric constant of the substrate, c is the speed of light in free space and is equal to 3 × 10^8^ m/s, A is area of the patch and r (=R1) is the radius of the patch.

**Figure 2:**
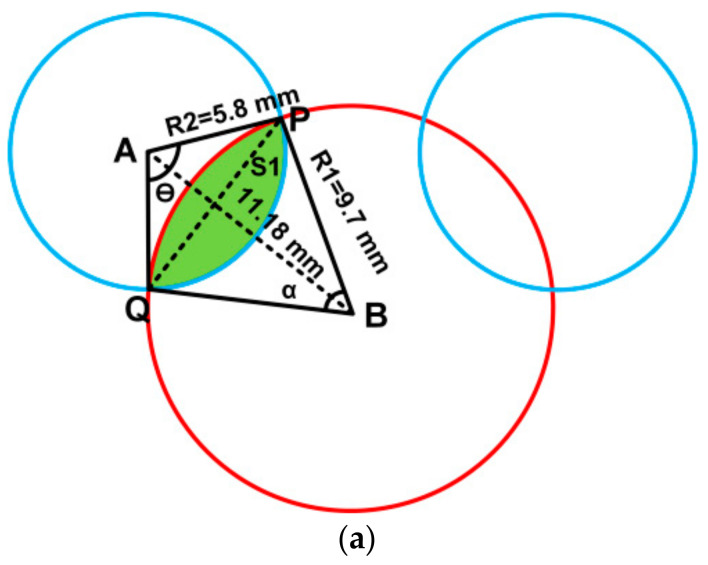

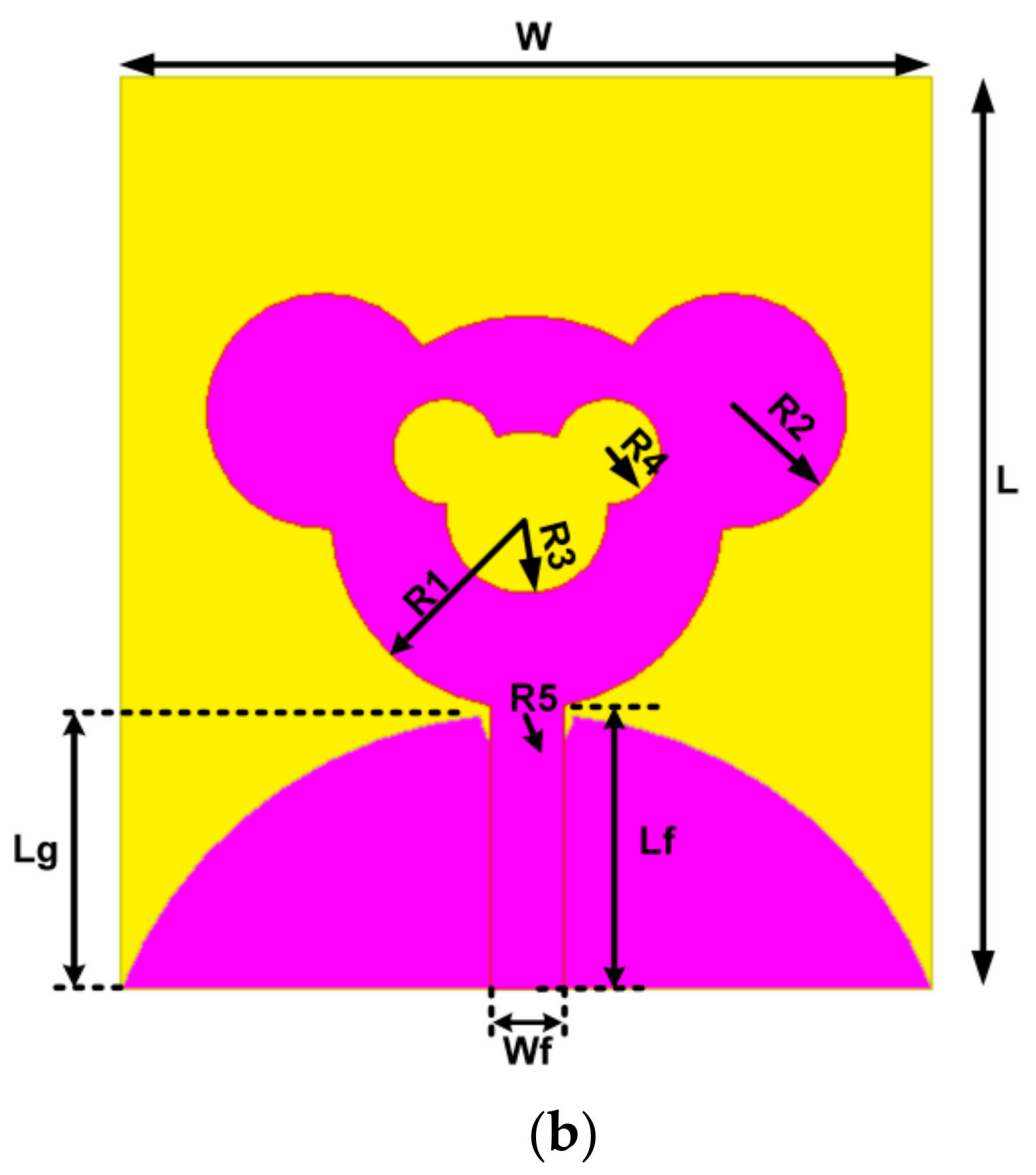
(**a**) Designed geometry of proposed radiator used for area calculations for outer ears. (**b**) Design geometry of proposed radiator.

**Figure 3:**
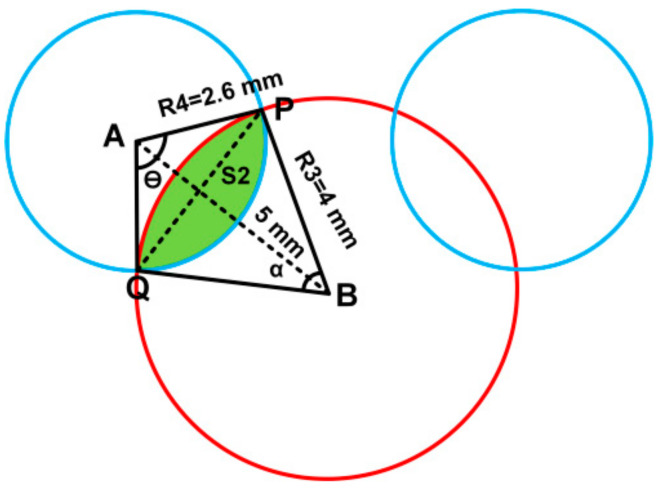
Designed geometry of proposed radiator used for area calculations for inner ears.

The area of the circle with radius R1, R2, R3 and R4 can be calculated from Figure 2 and Figure 3 by the following equations.

Area of R1, $A_{R1}=\pi \times R_{1}{}^{2}=295.6{\;\mathrm{mm}}^{2}$
Area of R2, $A_{R2}=\pi \times R_{2}{}^{2}=105.68{\;\mathrm{mm}}^{2}$
Area of R3, $A_{R3}=\pi \times R_{3}{}^{2}=50.26{\;\mathrm{mm}}^{2}$
Area of R4, $A_{R4}=\pi \times R_{4}{}^{2}=21.23{\;\mathrm{mm}}^{2}$

Area of shaded region S1:

Overlap area for the above shaded region (S1) can be found as follows.

(3) $$A_{S1}=\frac{1}{2}R_{2}{}^{2}(\theta -\sin\theta )-\frac{1}{2}R_{1}{}^{2}(\alpha -\sin\alpha )$$

where

$$\theta =2\times \sin^{-1}\left(\frac{9.7}{11.18}\right)=2.1\mathrm{radians}\;\alpha =2\times \sin^{-1}\left(\frac{5.8}{11.18}\right)=1.09\mathrm{radians}$$

The shaded region area,

$$\begin{array}{l} As_{1}=\frac{1}{2}{\left(5.8\right)}^{2}\left[\theta -\sin\left(\theta \right)\right]-\frac{1}{2}{\left(9.7\right)}^{2}\left[\alpha -\sin\left(\alpha \right)\right]\\ As_{1}=20.8-9.86=11.23{\;\mathrm{mm}}^{2} \end{array}$$

To find the effective area of R2:

$$Area\;of\;R2-Overlap\;area\;S1=105.68-11.23=94.45{\mathrm{mm}}^{2}$$

To find the area of slot:

The shaded region (S2) area can be found as follows,

$$\theta =2\times \sin^{-1}\left(\frac{4}{5}\right)=1.854\mathrm{radians}\;\alpha =2\times \sin^{-1}\left(\frac{2.6}{5}\right)=1.093\mathrm{radians}\;\begin{array}{l} As_{2}=\frac{1}{2}{\left(2.6\right)}^{2}\left[\theta -\sin\left(\theta \right)\right]-\frac{1}{2}{\left(4\right)}^{2}\left[\alpha -\sin\left(\alpha \right)\right]\\ As_{2}=3.02-1.64=1.38{\;\mathrm{mm}}^{2} \end{array}$$

Now, the effective area of R4 can be found as follows.

$$Area\;of\;R4-Overlap\;Area\;S2=21.23-1.38=19.85{\mathrm{mm}}^{2}$$

Thus, the area of slot can be calculated as,

$$\begin{array}{ll} Area\;of\;slot & =Area\;of\;R3+2\times 19.85\\ & =50.26+2\times 19.85\\ & =89.96{\mathrm{mm}}^{2} \end{array}$$

Now, the effective area of patch (A) can be found as,

$$Area\;of\;R1+Area\;of\;R2-Slot\;Area=295.6+2\times 94.45-89.96=394.54{\mathrm{mm}}^{2}$$

In addition, we know that for circle (*r* is the radius R1, *C* is the circumference of the circle).

(4) C=2⋅π⋅r

(5) $$A=\pi \cdot r^{2}$$

(6) $$C=2\cdot \frac{A}{r}$$

(7) $$f_{L}=\frac{c}{2\times C\sqrt{\varepsilon_{eff}}}$$

$$\begin{array}{l} f_{L}=\frac{c}{2\times 2\cdot \frac{A}{r}\sqrt{\varepsilon_{eff}}}\\ f_{L}=\frac{3\times 10^{11}}{2\times 2\cdot \frac{394.54}{9.7}\sqrt{2.095}}=1.273GHz \end{array}$$

From the above calculations, we can conclude that the derived equation for $f_{L}$ corresponds to the lower cut-off frequency for the patch. The theoretically calculated value of $f_{L}$ and the one obtained from the HFSS simulation are in close proximity with each other.

### 3.1. Effect of Shape of Radiator and Ground Plane

**Figure 4:**
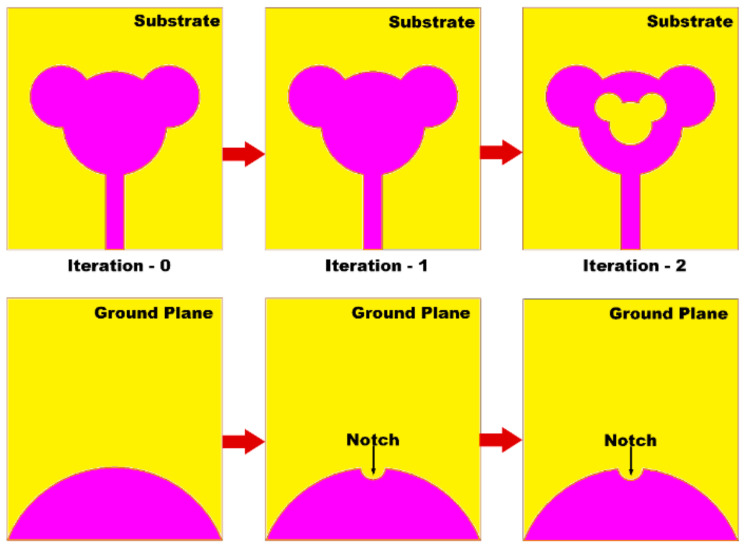
Deriving stages of the concentric Mickey-Mouse-shaped super wideband antenna.

**Figure 5:**
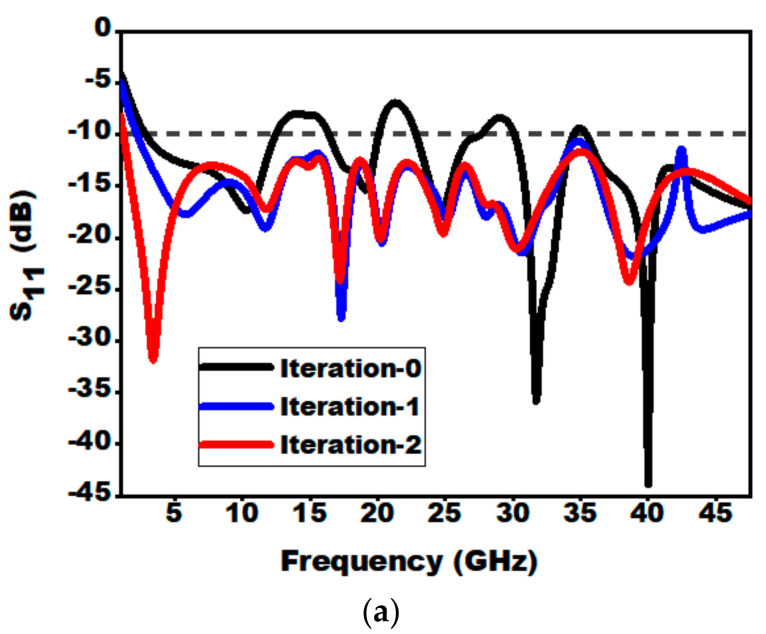

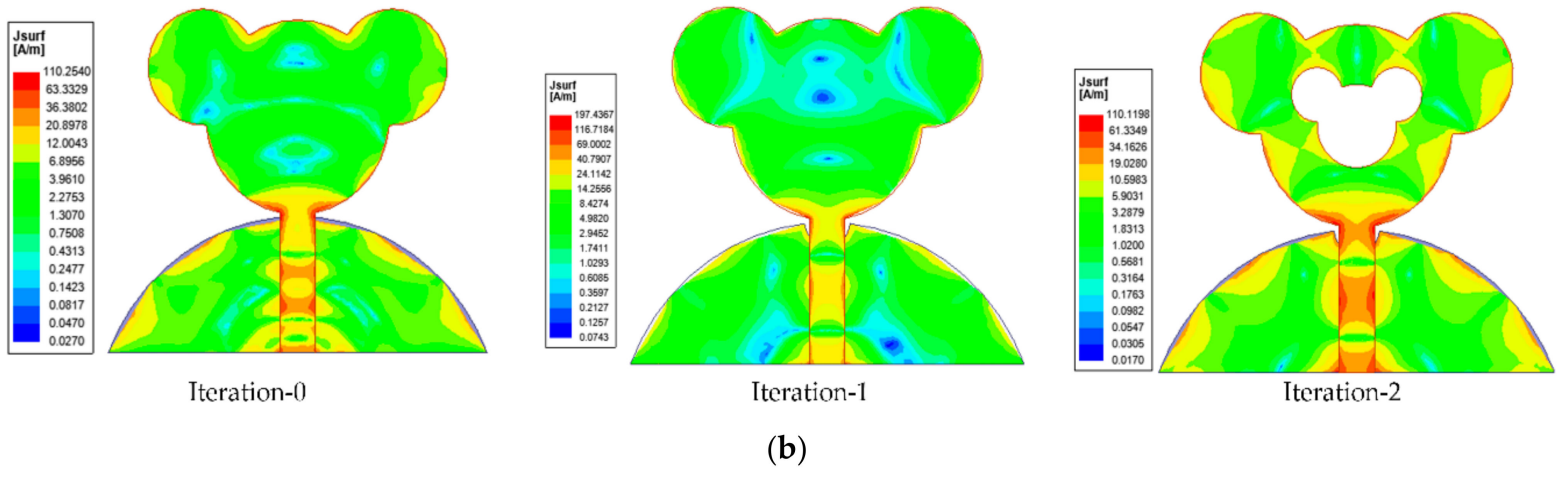
(**a**) Change of S_11_ with frequency for different iterations of the presented radiator. (**b**) Surface current distribution for Iteration-0, Iteration-1 and Iteration-2 at 15 GHz.

The derived phases of the proposed structure are presented in Figure 4. The resemblance between the reflection coefficients (S_11_) of the designed antenna structures at individual derived stages has been demonstrated in Figure 5. In case of zeroth iteration, it is worth noting that impedance matching is not so well over the entire frequency because of abrupt variation in impedance at the intersecting point of the radiator and the feedline. Hence, in iteration 1, to minimize the effect of capacitance, a notch is inserted in the semi-elliptical ground plane. As the coupling is minimized, there is a depletion in overall capacitance, which as a result increases impedance bandwidth. Furthermore, in the case of the second iterative structure, the introduction of slot in the top portion of the monopole augments the electrical length of monopole and lowers the cut-off frequency from 2.2 to 1.22 GHz, which in turns enhances the BDR as shown in iteration 2. Thus, it can be observed that, compared to iteration 0, iteration 2 assists in attaining better impedance matching over the entire frequency range from 1.22 to 47.5 GHz. The aforementioned analysis can be further concluded by the study of surface current distribution at 15 GHz (i.e., as at this frequency the S_11_ value of iteration 0 is above 10 dB, while for iteration 1 and 2 it improves. The same explanation can be extended for other frequencies at which the value of S_11_ goes above −10 dB for iteration 0. From Figure 5b, it can be seen that for iteration 0 and 1, the surface current is distributed over the entire patch and feed line. Whereas for iteration 2, the surface current is concentrated near the feedline which confirms that the impedance matching is good.

### 3.2. Parametric Analysis

In order to enhance the performance of designed antenna, parametric analysis is performed to optimize the dimensions of different parameters. The effects of different parameters on the performance of the antenna are presented in this section.

#### 3.2.1. Effect of Position of Outer and Inner Ears

**Figure 6:**
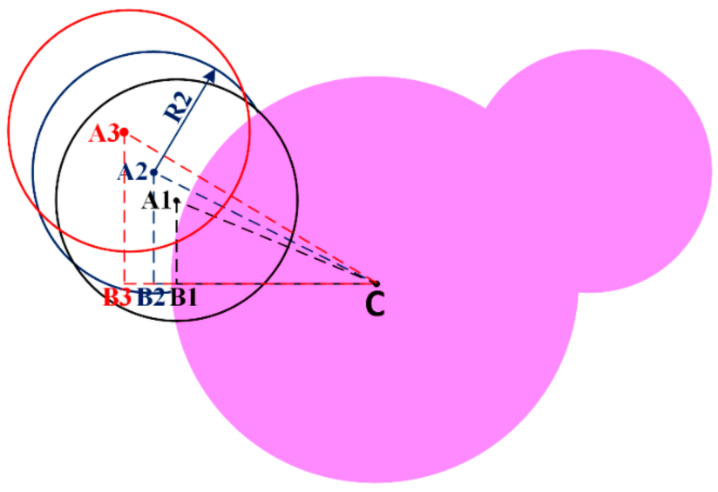
Position of the outer ear with respect to the center of the patch.

**Figure 7:**
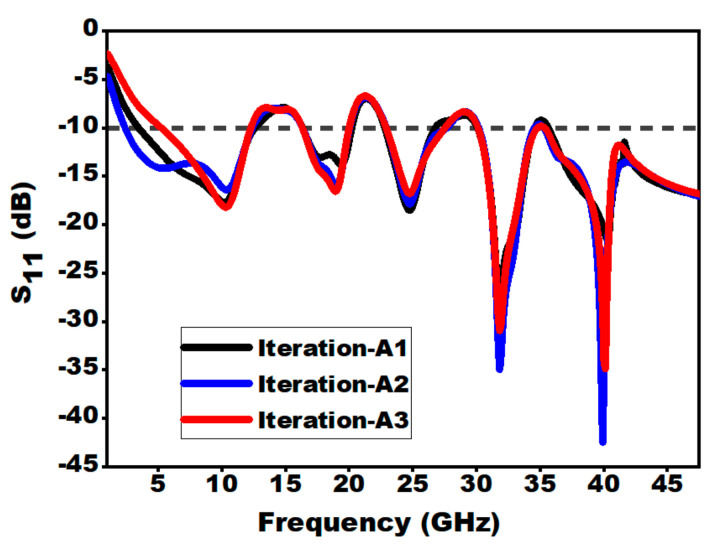
S_11_ for different positions of outer ears.

A parametric study on the position of the inner and outer ears is carried out in order to achieve the optimum outcome for extending the bandwidth of the patch antenna. The position of the ears has been optimized for getting the wider bandwidth and better impedance matching by iterating through various positions and assorted distance from the center of the patch. Figure 6 illustrates the position for outer ears with respect to the center of the patch. Table 2 points out the different segment length values for different positions of the outer ear. From Figure 7, it can be seen that iteration A2 upholds the position for the outer ears, signifying better bandwidth. The lower cut-off frequency for A1 is 3.45 GHz, A2 is 2.4 GHz and A3 being 5.25 GHz. Hence, the A2 position gives the better lower cut-off frequency as compared to A1 and A3.

**Figure 8:**
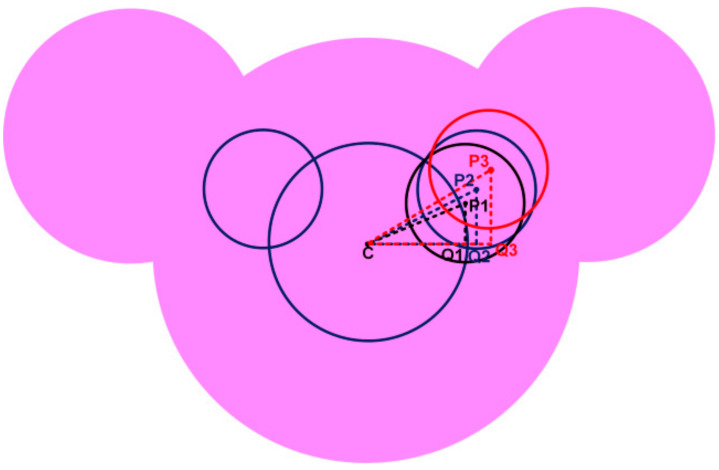
Position of inner ear with respect to the center of the patch.

**Figure 9:**
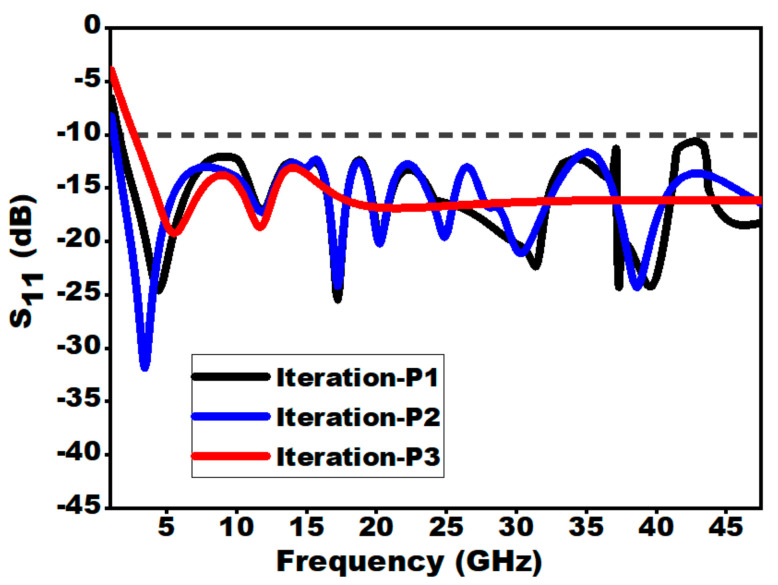
S_11_ for different positions of inner ears.

(a) Position of the Outer Ears:

Similarly, Table 3 highlights different segment length values for different positions of inner ears, illustrated in Figure 8. From Figure 9, it can be observed that iteration P2 justifies the position for the inner ears enhancing the lower frequency bandwidth and providing good impedance matching throughout the operating frequency. The lower cut-off frequency for P1 1.58 GHz, P2 1.22 GHz and P3 is 2.7 GHz.

**Figure 10:**
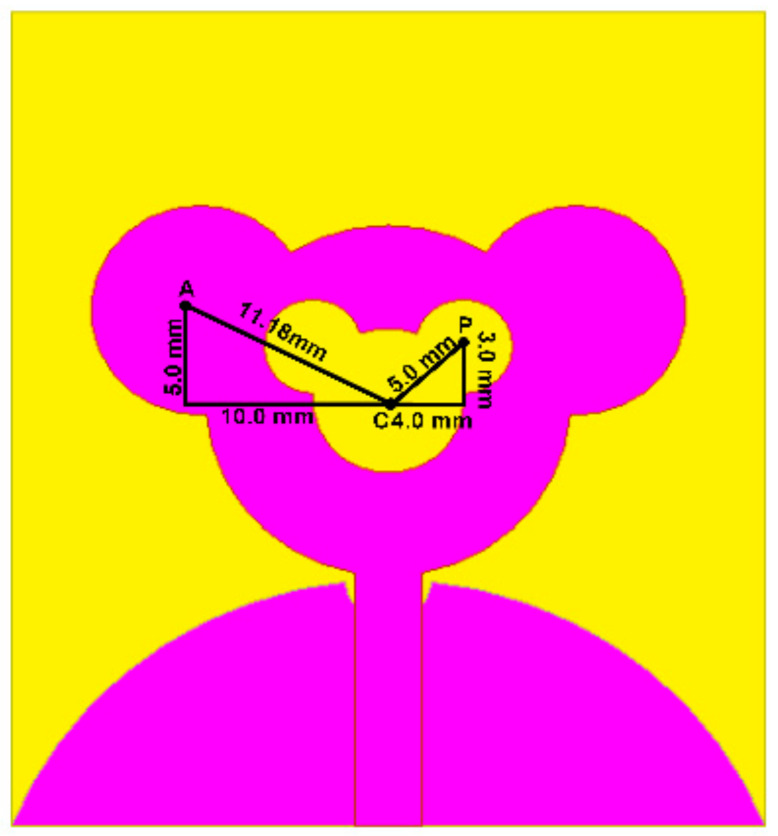
Placement of outer ears and inner ears of the antenna.

(b) Position of the Inner Ears:

The above analysis confirms that the position of inner and outer ears plays an important role in defining the impedance bandwidth and thus provides SWB characteristics. The detailed dimension layout of inner and outer ear for the proposed antenna is illustrated in Figure 10. However, the designer can use this as a preliminary guideline for defining the position and can come up with other alternative solutions.

#### 3.2.2. Effect of Feed Width (Wf)

**Figure 11:**
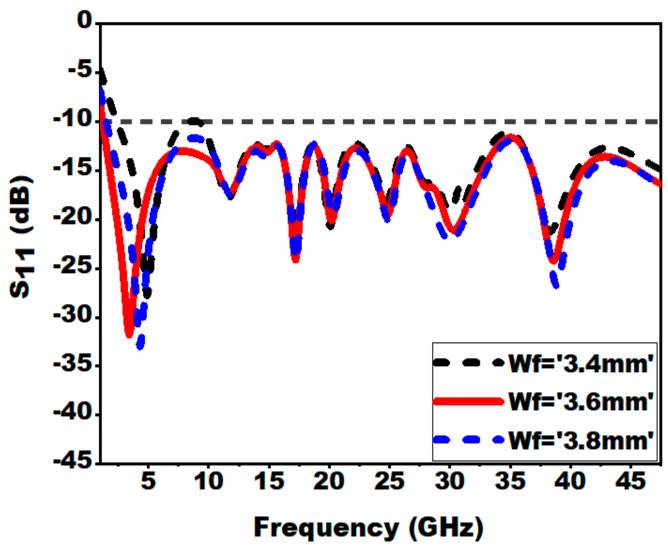
Effect of variation of feed width Wf on S_11_.

The study of the variation of the feed width is performed to attain wide impedance matching. The analysis is carried out by varying Wf from 3.4 mm to 3.8 mm in steps of 0.2 mm while retaining other design dimensions undisturbed. From Figure 11, it can be confirmed that improved impedance matching and good bandwidth is exhibited for Wf = 3.6 mm.

#### 3.2.3. Effect of Feed Length (Lf)

**Figure 12:**
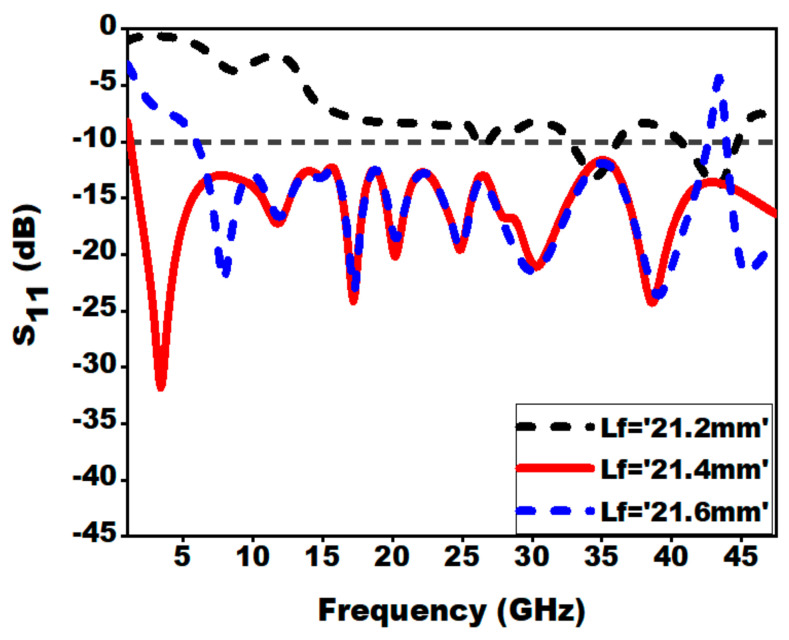
Effect of variation of feed length Lf on S_11_.

To fulfill the need of good impedance matching, the analysis is carried out by varying feed length by 0.2 mm. The study shows that with the variation of feed length, the lower resonance frequency is drastically effected. From Figure 12, the best impedance matching is observed at Lf = 21.4 mm. A microstrip feedline is utilized to acquire an impedance matching of 50 Ω.

#### 3.2.4. Effect of Presence of Notch on the Ground Plane (R5)

**Figure 13:**
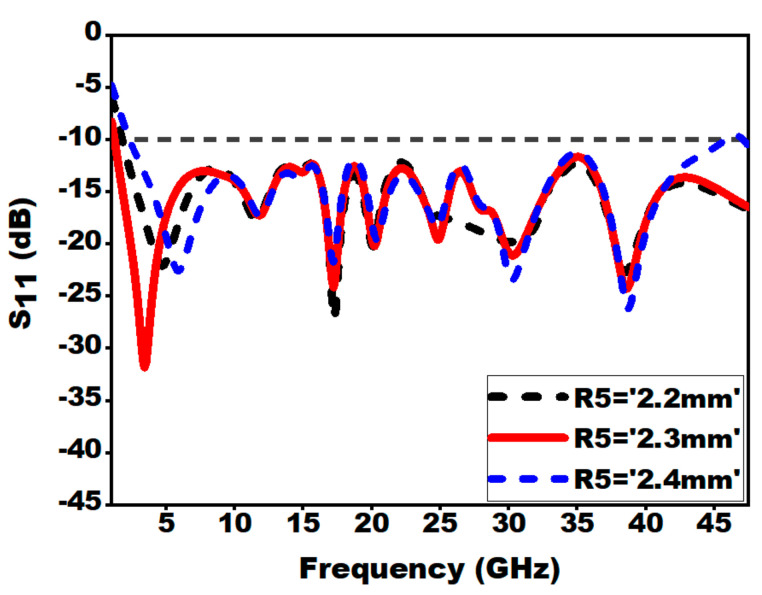
Effect of variation of notch radius (R5) on S_11_.

As presented in Figure 13, it is understandable that the notch on the ground plane is essential in enhancing the impedance bandwidth. This is due to the fact that their exists an electromagnetic coupling between the patch and the slotted ground plane. The inductive effect is neutralized by utilizing the slotted partial ground plane, as a result of which, the antenna structure impedance becomes purely resistive.

#### 3.2.5. Effect of Slot on Radiator (R3 and R4)

By loading slot on the radiator, lower cut-off frequency is shifted from 2.2 to 1.22 GHz. The introduction of slot on the patch increases the electric current path length. This technique enhances the electrical dimension of the concentric Mickey-Mouse-shaped patch and reduces the lower cut-off frequency.

**Figure 14:**
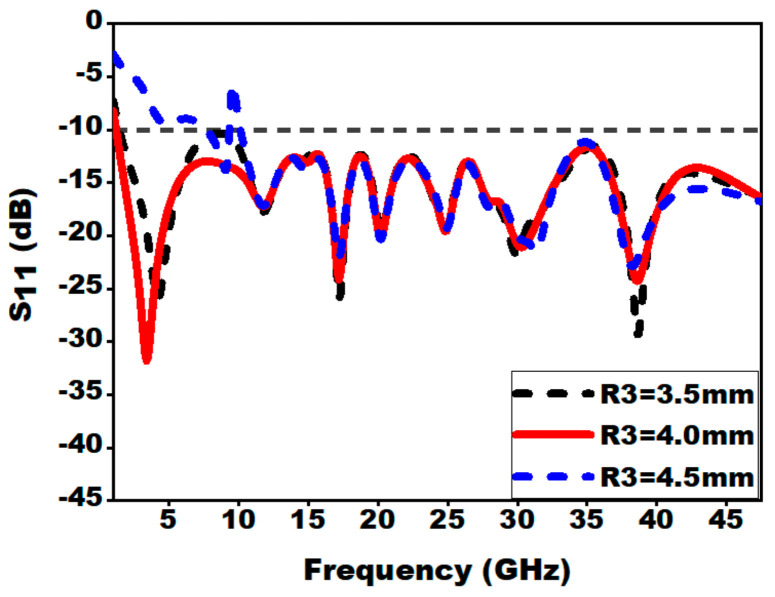
Effect of variation of inner radius (R3) on S_11_.

**Figure 15:**
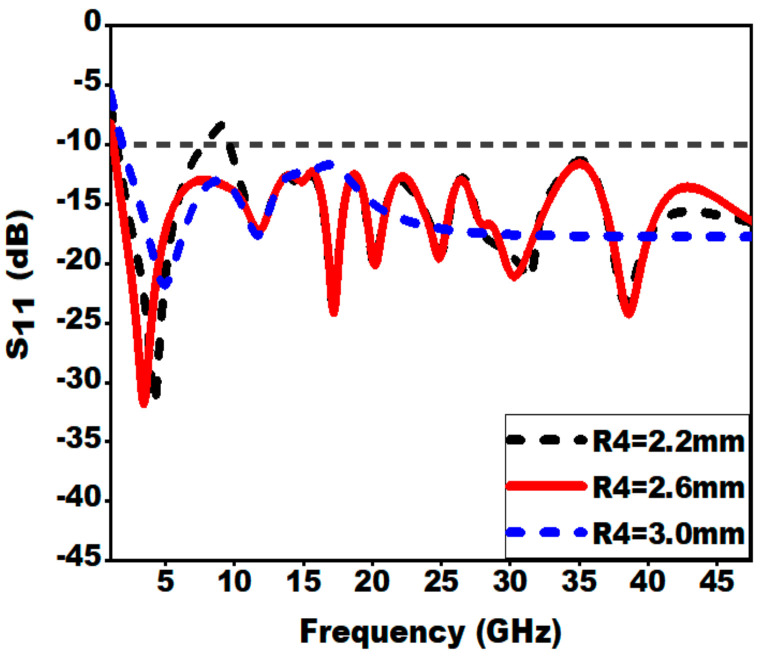
Effect of variation of inner radius (R4) on S_11_.

The effect of slot on radiator is shown in Figure 14 and Figure 15. From Figure 14, it can be seen that the optimal value of the radius R3 is 4.0 mm for which the lower cut-off frequency is moved to 1.22 GHz. A sharp glitch in the reflection coefficient is observed at 9.6 GHz for the R3 value of 4.5 mm; this is due to the fact that the surface current is distributed around the edges of the ground plane and edges of the slot, which is not perfectly matched to 50 Ω.

#### 3.2.6. Effect of Outer Radius of the Patch (R1 and R2)

**Figure 16:**
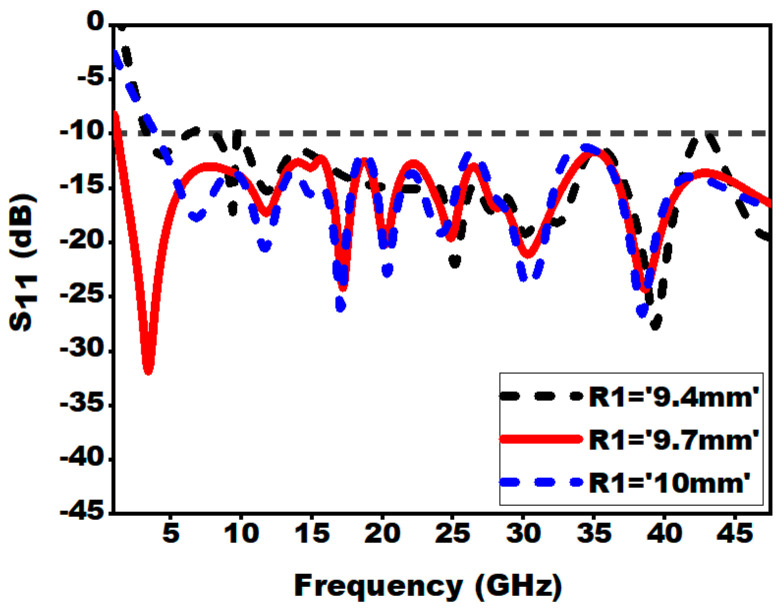
Effect of variation of outer radius (R1) on S_11_.

**Figure 17:**
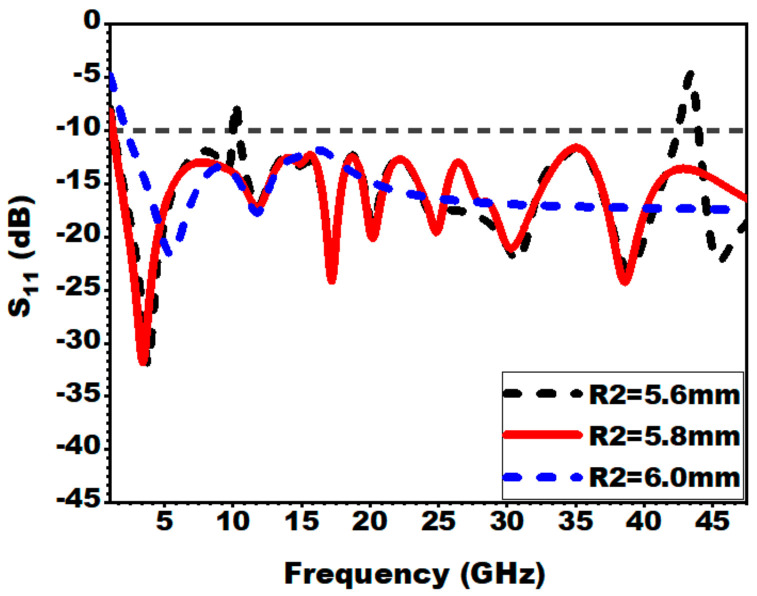
Effect of variation of outer radius (R2) on S_11_.

Figure 16 and Figure 17 depict the influence of the variation of the outer radius of the patch on S_11_. It can be noted that altering the radius of the patch from a known value leads to alleviation of impedance bandwidth. For the presented antenna design, the outer radius of R1 = 9.7 mm and R2 = 5.8 mm is used as the optimized values.

#### 3.2.7. Effect of Ground Plane Length (Lg)

**Figure 18:**
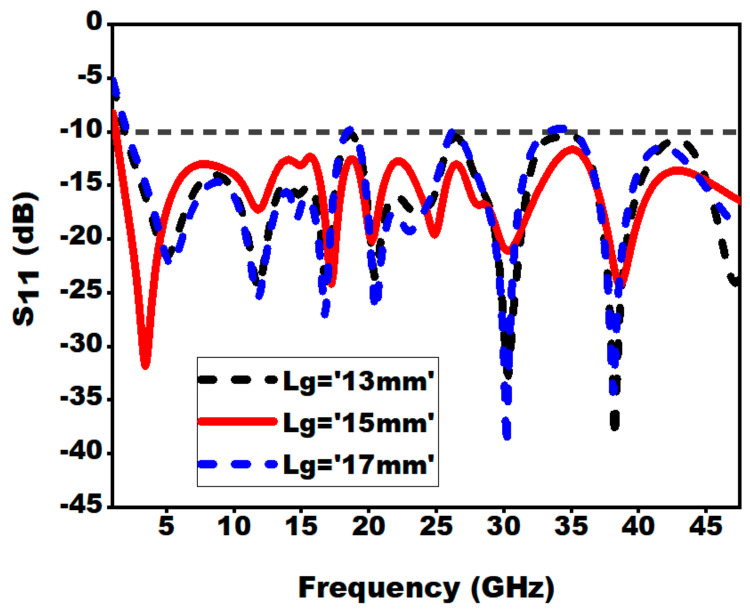
Effect of variation of ground length (Lg) on S_11_.

The length of the ground plane is an essential metric in the SWB antenna design. From Figure 18, it can be clearly pointed out that impedance bandwidth is influenced by the length of the ground plane. An increase or decrease in the length of ground plane results in an increase or decrease in the current flow on the upper portion of ground plane. This leads to a decrease or increase of inductance of the antenna; therefore, the resonance mode location gets shifted upwards or downwards.

### 3.3. Surface Current Distribution

**Figure 19:**
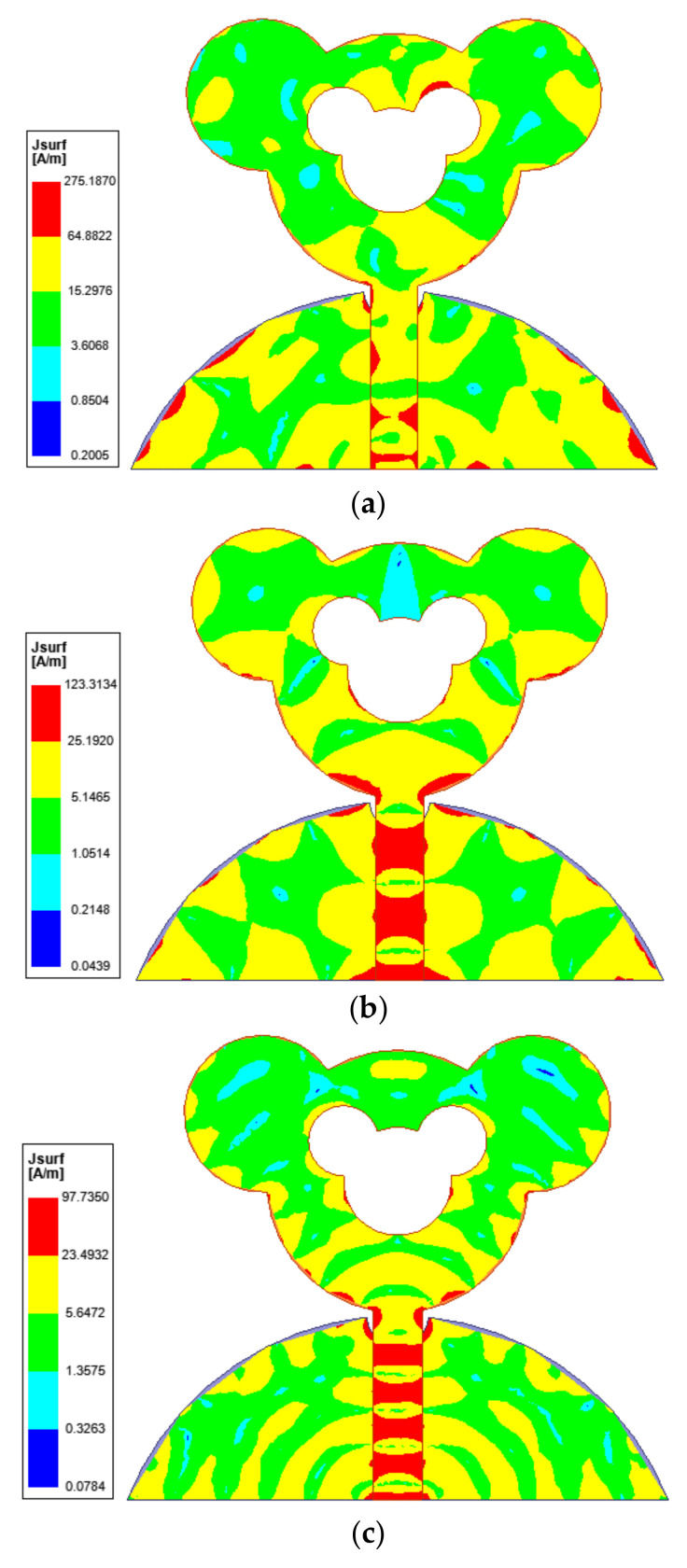
Simulated surface current distribution on designed antenna at low, mid and high resonance frequency: (**a**) 3.4 GHz, (**b**) 20.2 GHz, (**c**) 38.6 GHz.

For the purpose of better understanding the resonance behavior of the designed antenna, its surface current distribution at low, mid and high frequency is studied and illustrated in Figure 19. The density of current is maximum in the feed line, edges of the modified patch and edges of the ground plane. The current distribution pattern at the first resonance at 3.4 GHz implies first-order harmonic mode. The current distribution pattern at the mid resonance at 20.2 GHz indicates a higher harmonic mode and so on. In designing wideband antenna, to achieve a broad frequency spectrum, it is necessary to superimpose neighboring modes on one another. The presented antenna attains the SWB functionality given that the surface current preserves a harmonic order flow in the radiating slotted partial ground plane.

## 4. Modelling of Equivalent Circuit for the Presented Antenna

**Figure 20:**
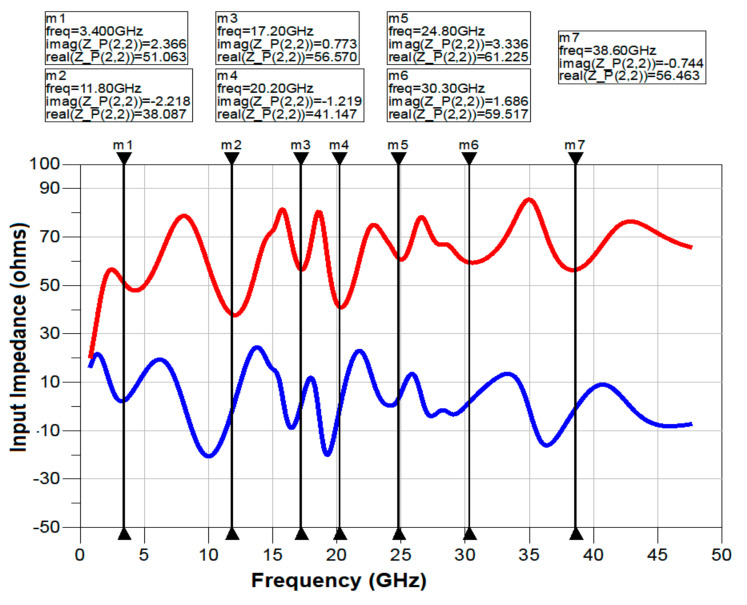
Real and imaginary parts of input impedance versus frequency.

**Figure 21:**
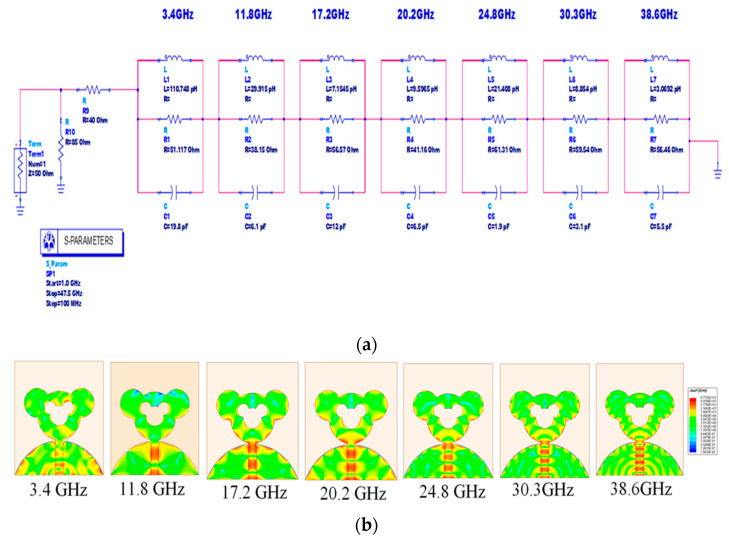
Equivalent circuit model and current distribution of antenna; (a) equivalent circuit model as implemented in ADS, (**b**) Mapping of surface current distribution for each resonance frequency.

The equivalent circuit model of the designed antenna is implemented using ADS, as shown in Figure 20 and Figure 21. The reflection coefficient response has been used in order to derive the equivalent circuit model. Each fall off below −10 dB in the reflection coefficient is represented by an equivalent resonant circuit model using RLC elements. As indicated in Figure 20, there are seven resonant modes observed in the shown frequency band from 1.2 to 47.5 GHz. The markers from m1 to m7 indicate the real and imaginary impedances for the seven resonant frequencies. The value of the real part of the impedance is close to 50 Ω while the imaginary is close to 0 Ω, which signifies the proper resonances at these points.

The equivalent model derived from the data points of the input impedance is shown in Figure 21. The lumped component values of the equivalent circuit, i.e., R, L, C, can be calculated initially by using Equations (8)–(10) [28,29] considering the bandwidth and resonant frequency matching to 50 Ω. The values are tabulated in Table 4. The lumped component values are tuned a little bit by using ADS software to achieve desirable SWB characteristics. The impedance is represented by seven parallel RLC cells that are connected in series and they resonate at respective resonant frequencies.

(8) $$L=\frac{{\mathrm{imag}(Z}_{11})}{2\pi f}$$

(9) $$C=\frac{1}{{\left(2\pi f\right)}^{2}L}$$

(10) $$f=\frac{1}{2\pi \sqrt{LC}}$$

**Figure 22:**
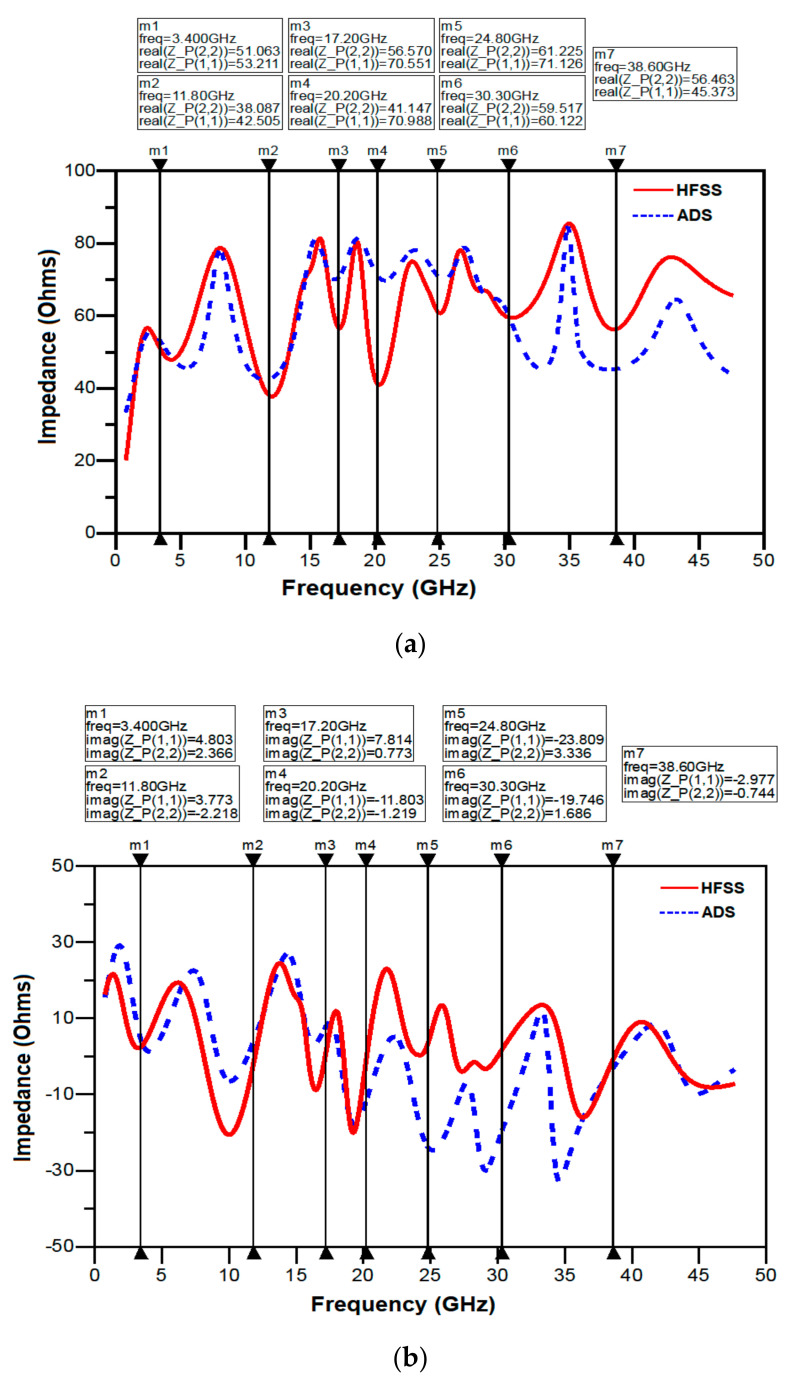
Variation of input impedance versus frequency; (**a**) real part, (**b**) imaginary part.

Figure 22 shows the variation of input impedance with respect to frequency. From Figure 22, it can be seen that there is good match between the results obtained from HFSS and ADS at seven designated resonant frequencies. However, at higher frequencies there is a deviation in the values, because of the fact that the equivalent circuit model is approximately matched to 50 Ω.

**Figure 23:**
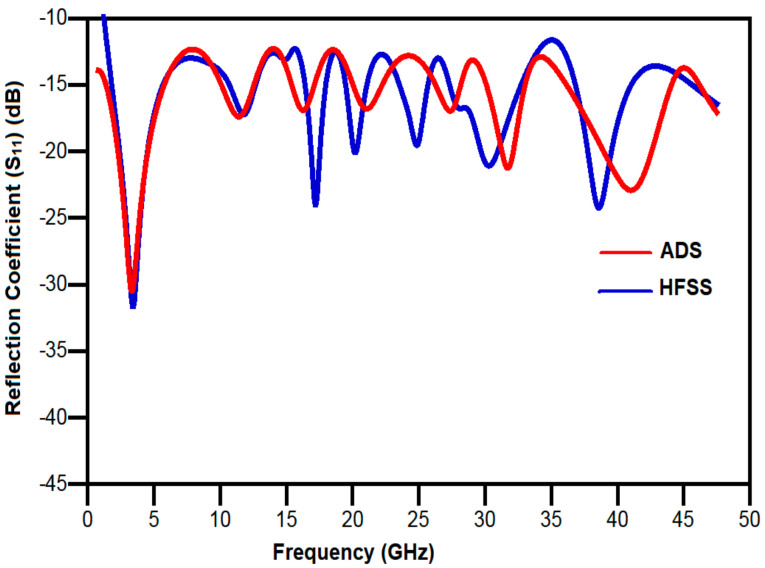
Comparison of reflection coefficient obtained from HFSS and ADS.

Figure 23 shows the comparison between the reflection coefficients obtained from HFSS and ADS. As indicated in the figure, there is a small shift between the results obtained from the HFSS simulation and ADS. This shift can be attributed to the capacitors; inductors and resistors used in the ADS simulation were adjusted to satisfy the proper response which causes a shift in resonance frequencies from the HFSS simulation results.

From Figure 21, it is observed that the surface current is perceived around the feed line and the patch for respective resonances. From the magnitude of the current and voltage, it can be seen that the impedance is centered along the feed line and edges of the patch for each of the resonating frequencies.

Thus, from the above discussion, it may be concluded that the equivalent circuit model provides good insight into the details of resonant frequencies and its prominence with respect to the input impedance.

## 5. Results and Discussions

**Figure 24:**
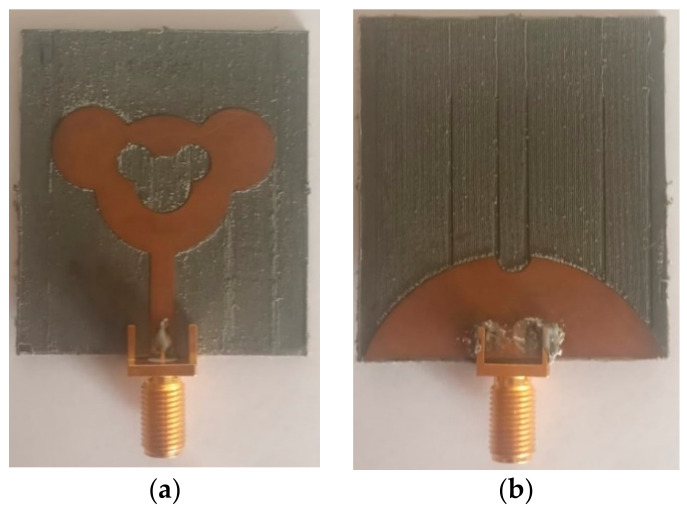
Photograph of fabricated super wideband (SWB) Antenna. (**a**) Front part; (**b**) Ground palne.

This section concentrates on the comparison of experimental and simulated results. The presented design in Figure 2 is simulated on HFSS v.19.0 using FEM method. The optimized dimensions of the SWB concentric Mickey-Mouse-shaped monopole achieved through parametric studies are tabulated in Table 1. The image of fabricated antenna prototype with top and bottom view is shown in Figure 24a,b, respectively. The model number of SMA connector used is SMA202-2. It is an SMA female for PCB Board Edge Soldering RF Connector. Its operating frequency is from DC to 18 GHz with 50 Ω matched.

By making use of these dimensions, a prototype was developed and has been measured by Anritsu 37269A Vector Network Analyzer, 40 MHz–40 GHz. Even though the designed antenna functions well for the frequency spectrum of 1.22–47.5 GHz, the experimental results are measured only up to 40 GHz due to the higher frequency limitation of the available VNA.

**Figure 25:**
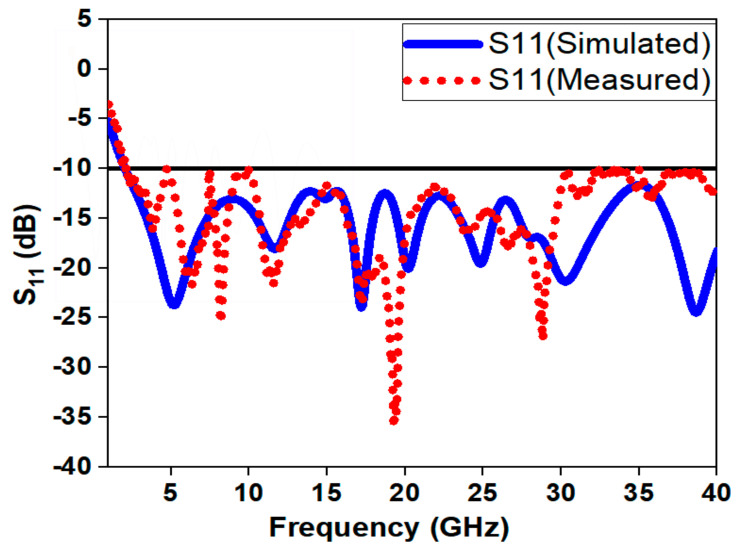
Reflection coefficient characteristics of presented antenna.

From the Figure 25, we can observe that the measured result for the reflection coefficient (S_11_) is in close proximity with the simulated one for the frequencies from 1 to 30 GHz and frequencies from 30 to 40 GHz; it is close to the −10 dB line. The reason for this is that the SMA connector being used supports frequencies up to 26.5 GHz and for higher frequencies its performance deteriorates. In addition, the small deviations in the result may be because of the widened ground effect due to SMA connectors, which is not included in the simulation environment.

Additionally, it may be due to substrate material tolerance as the ideal substrate specifications that are used in HFSS environment may not be feasible in the practical environment. From Figure 25, it can be noticed that the resonance occurs at 3.4/11.8/17.2/20.2/24.8/30.3 and 38.6 GHz in simulation and at 3.83/6.34/8.19/11.55/17.11/19.28/28.3 GHz in measurement. The designed antenna provides a good impedance bandwidth for the entire frequency spectrum of 1.22–47.5 GHz with a fractional bandwidth of 190% and bandwidth ratio of 39.5:1. The presented antenna has the highest BDR of 6895. The measured frequency range is from 1.25 to 40 GHz with fractional bandwidth of 188% and BDR of 6523. A wideband antenna with compact structure substantiates a high value of BDR. In addition, the BDR signifies two prominent factors, i.e., miniaturization and wide bandwidth. The expression for BDR can be given in Equation (11).

(11) $$BDR=\frac{BW\%}{\lambda_{length}\times \lambda_{width}}$$

where $\lambda_{width}$ and $\lambda_{length}$ are the wavelength associated with the lower cut-off frequency.

**Figure 26:**
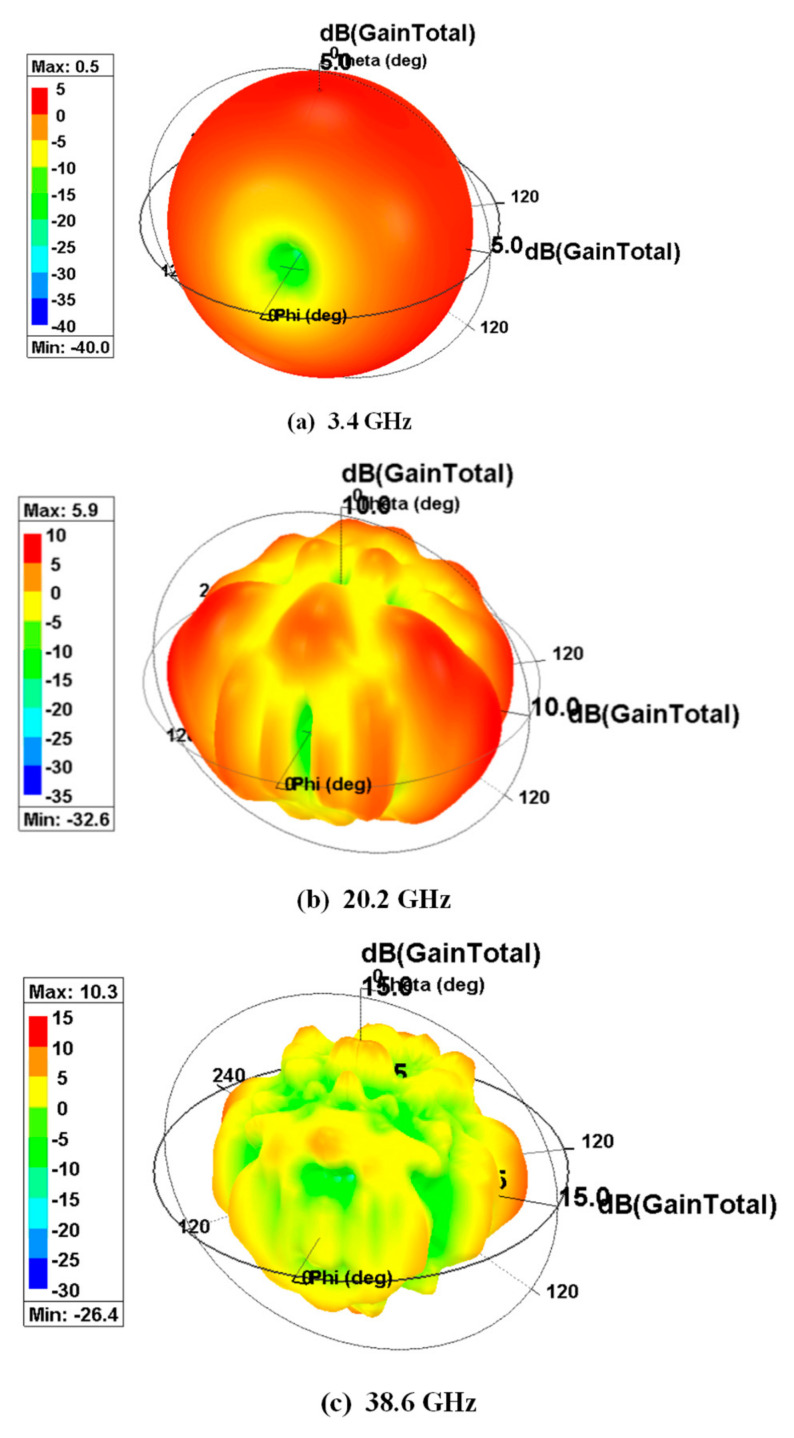
Three-dimensional polar gain plot for the presented antenna at (**a**) 3.4 GHz, (**b**) 20.2 GHz, (**c**) 38.6 GHz.

The results of simulation with respect to 3D polar gain plot of the presented SWB antenna at low, mid and higher resonance frequencies at 3.4 GHz, 20.2 GHz and 38.6 GHz are presented in Figure 26. The pattern at 3.4 GHz (Figure 26a) is omnidirectional in nature with an overall gain of 0.5 dB. At mid frequency of 20.2 GHz (Figure 26b), the pattern is bidirectional in nature with minor back lobes where an overall gain of 5.9 dB is witnessed. Correspondingly, at a high resonance frequency of 38.6 GHz (Figure 26c), an overall simulated gain of nearly 10.3 dB is observed.

**Figure 27:**
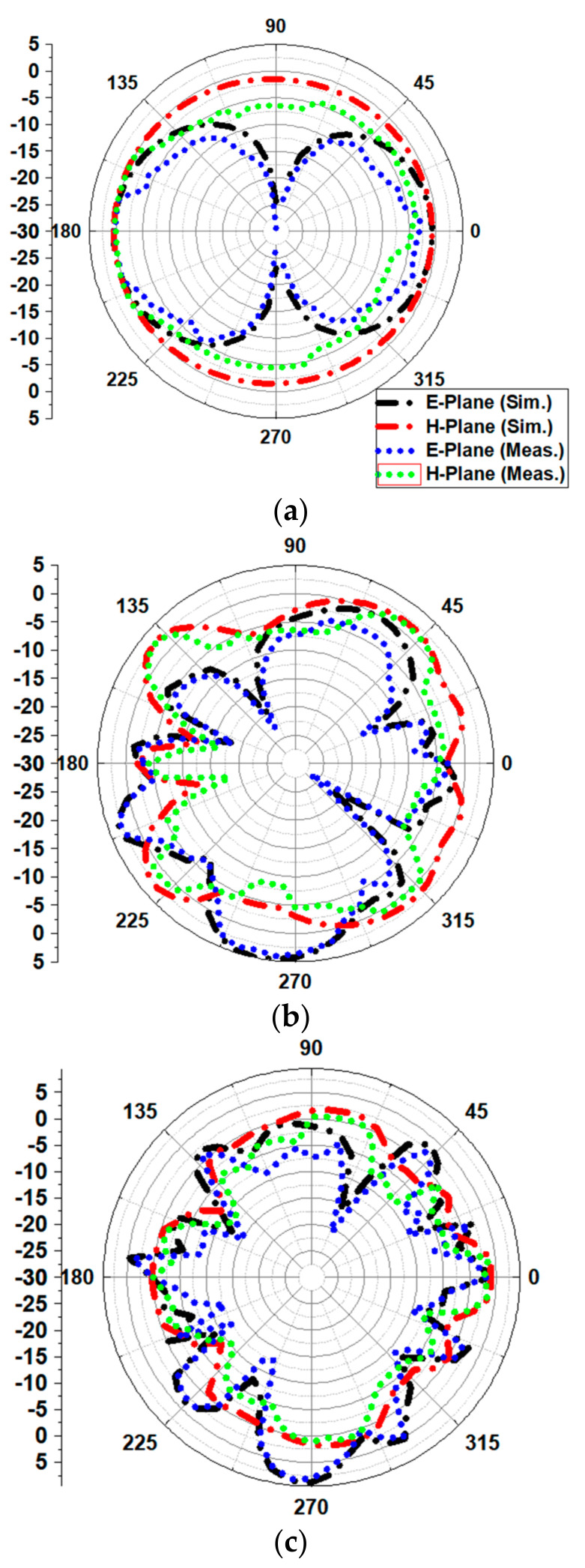
Simulated and measured far field radiation patterns of presented radiator in the E plane and H plane: (**a**) 3.4 GHz, (**b**) 20.2 GHz, (**c**) 38.6 GHz.

**Figure 28:**
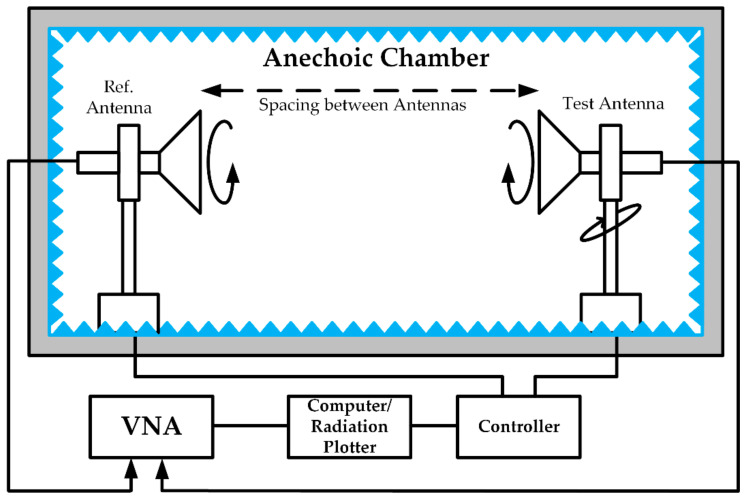
Schematic of anechoic chamber with control accessories.

Figure 27 illustrates the radiation patterns in each of the planes, i.e., E plane (*ⲫ* = 0) and H plane (*ⲫ* = 90) at low, mid and higher resonance frequency. Automatic anechoic chamber is used to measure the radiation patterns. The schematic of anechoic chamber is as shown in Figure 28. The radiation patterns are recorded for an angle of 5° as the stepper motor used in the control circuitry had provision to rotate by 5° only. The spacing between the reference antenna and test antenna was 3 m. From Figure 27, it can be concluded that the presented antenna demonstrates nearly an omnidirectional pattern in the H plane and bi-directional pattern in the E plane. With the increase in frequency, the patterns start attaining distorted omnidirectional nature in the E and H planes. At high frequencies, such distortions are because of the excitation of higher order modes.

At lower frequency, the current is uniformly spread on the ground plane and patch. Hence, the radiation patterns are omnidirectional in the H plane and bidirectional in the E plane. At higher frequencies, higher order modes are induced, and the current density is no longer evenly spread on the radiator. As a consequence, radiated modes may promote an increased level of electromagnetic interference. In order to suppress these unwanted radiated modes, designers can employ techniques such as introducing periodic structures, defected ground structure (DGS), parasitic elements, split ring resonators (SRRs) [29], etc. However, these techniques increase the complexity of the design and also the cost of fabrication.

**Figure 29:**
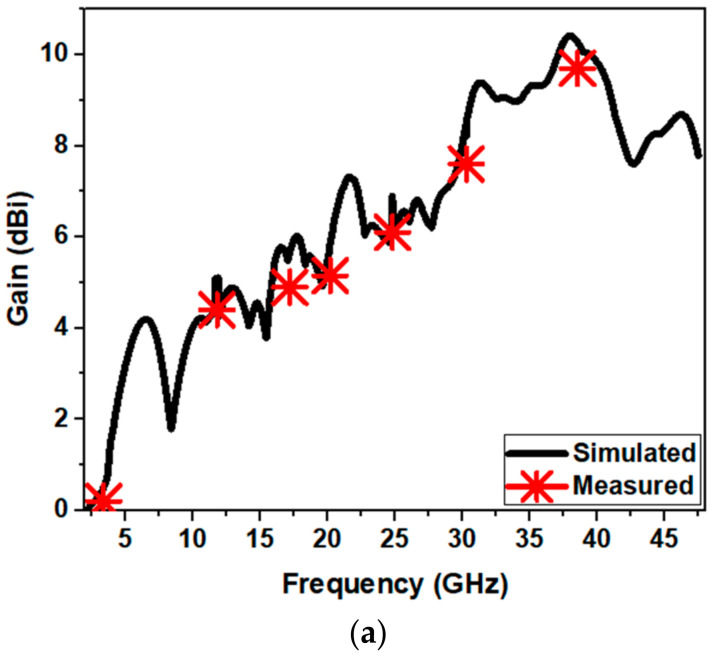

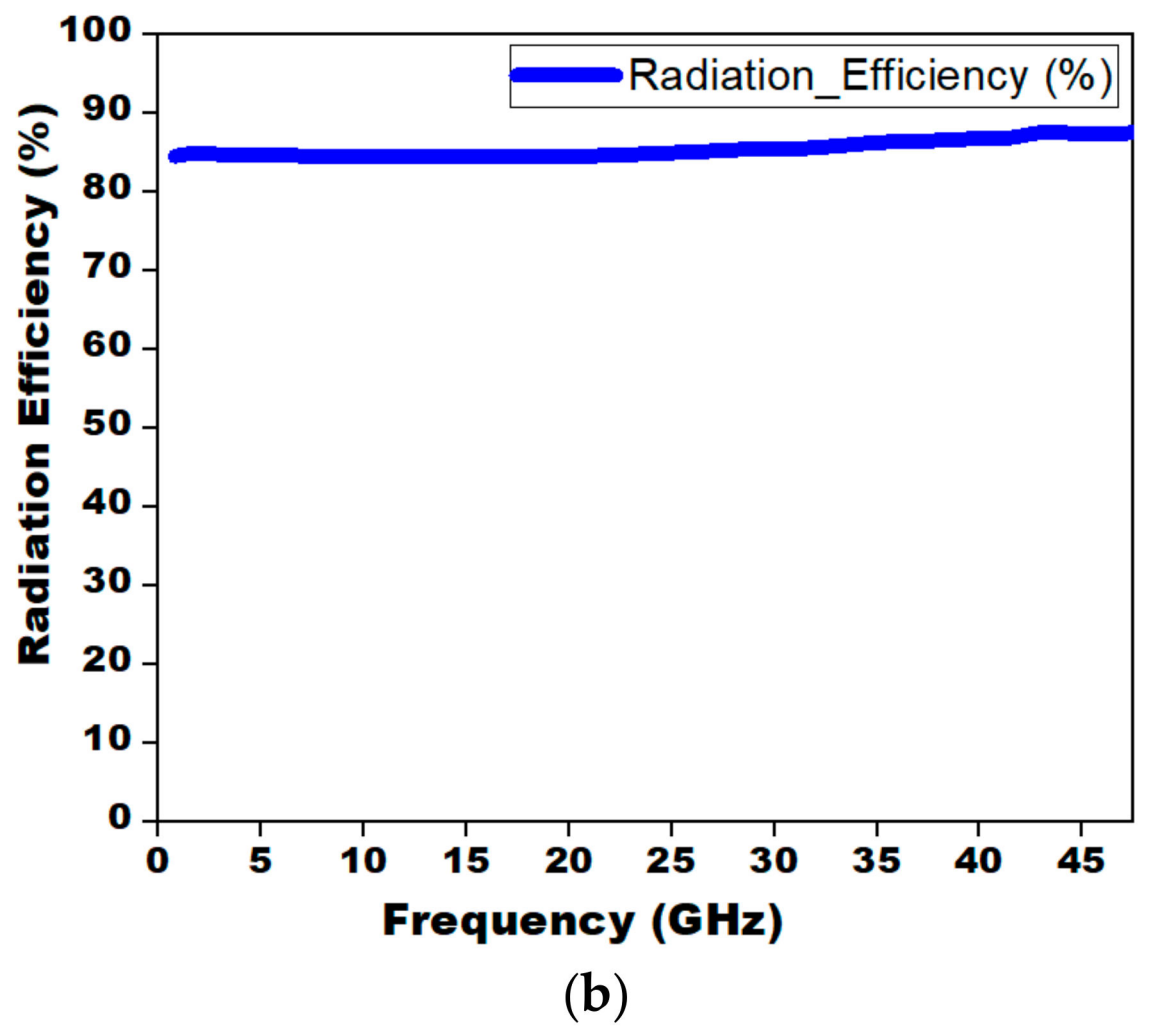
(**a**) Gain variation of proposed antenna and (**b**) simulated radiation efficiency vs. frequency.

Figure 29a demonstrates the gain (simulated and measured) variation of the proposed antenna. It is seen from the Figure 29a that, as the frequency increases, the value of the gain increases. Accordingly, the effective aperture of the antenna increases in relative to the wavelength. At the mid frequency range, the gain has slightly reduced and became flat. This is because of the fact that, at the sharp edges of the structure, the current intensity increases with respect to frequency, which causes delay paths in antenna and hence gain drops a little. It is also evident from Figure 21 that the current at 20.2 GHz is primarily on the ground plane, which signifies that there is a narrow band resonant structure at this frequency. Simulated gain varies from 0.5 to 10.3 dBi and measured gain varies from 0.2 to 9.7 dBi. Figure 29b depicts the simulated radiation efficiency of proposed antenna. It is observed that the radiation efficiency is increasing with respect to frequency and maximum efficiency of about 88% is observed at 42 GHz.

## 6. Time Domain Performance

**Figure 30:**
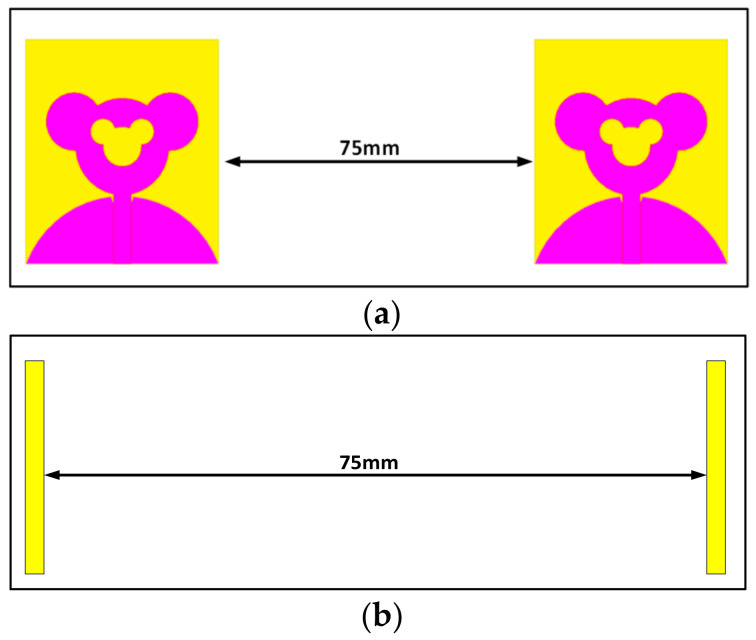
Time domain characterization arrangement for (**a**) side by side and (**b**) face to face.

**Figure 31:**
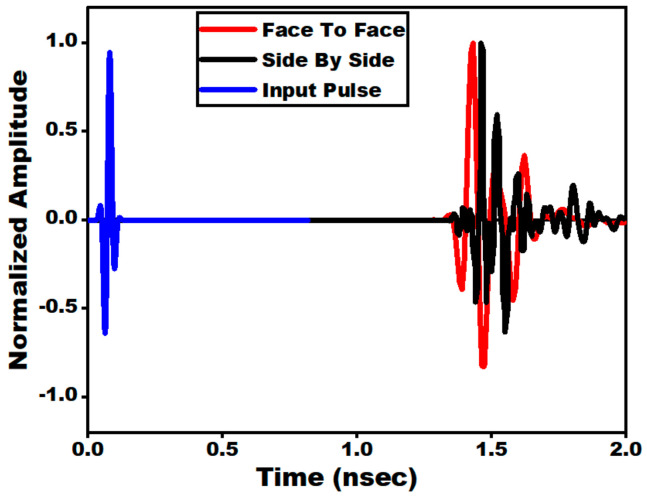
Simulated normalized amplitude of input and received signal pulse in side by side and face to face arrangement.

This time domain analysis is utilized to characterize pulse-shaping properties of the antennas. The duration of the pulses is in the range of about hundreds of pico-seconds. Ideally, the received SWB pulses should be of the same shape as the transmitted pulse. However, practically, the received pulses are skewed in shape and are prompt to a long tail which is termed as the “ringing effect”. Therefore, the antenna should be designed carefully to circumvent distortions. Hence, for wideband antennas, the time domain characterization is very much essential. Thus, in order to ensure the received pulse is the precise reproduction of the transmitted pulse, time domain analysis is performed. For carrying out the time domain analysis, two similar antennas are arranged at some distance. The distance between two similar antennas is 75 mm in each case. Two distinct configurations: (i) side by side and (ii) face to face are considered. The configurations are as presented in Figure 30a,b. For both the arrangements, Gaussian pulses are adopted for transmission and reception as depicted in Figure 31. With the aid of Figure 31, a significant time domain parameter can be computed, i.e., fidelity factor (FF), which represents the equivalence between the transmitted and received waveforms. It can be observed that, before the excitation begins, a small amount of ringing is detected in both the received pulses. This is due to the effect of channel noise. However, the pulse shape is intact in both the received pulses which indicates lossless reception of transmitted data.

The normalized transmission and reception signal pulses are given in Equations (12) and (13).

(12) Ts^(t)=Ts(t)∫−∞∞Ts(t)2dt12

(13) Rs^(t)=Rs(t)∫−∞∞Rs(t)2dt12 

The fidelity factor is given by Equation (14)

(14) $$FF=\underset{T}{\max}\int_{-\infty }^{\infty }\hat{T_{s}}(t)\hat{R_{s}}(t+\tau )dt$$

where $T_{s}(t)$ is the transmitted pulse waveform and $R_{s}(t)$ is the pulse at the receiving antenna port B. As a result of normalization, the value of FF lies in the middle of 0 and 1. FF value of 1 indicates that, without any system loss, the signal pulse being received is identical to the signal pulse being transmitted. Similarly, for value 0, it indicates that, due to system attenuation, the signal being received is entirely different from the signal being transmitted. However, for FF values below 0.5, the pulse being received becomes entirely unrecognized. Hence, high values of FF ensure low distortion in the received signal.

**Figure 32:**
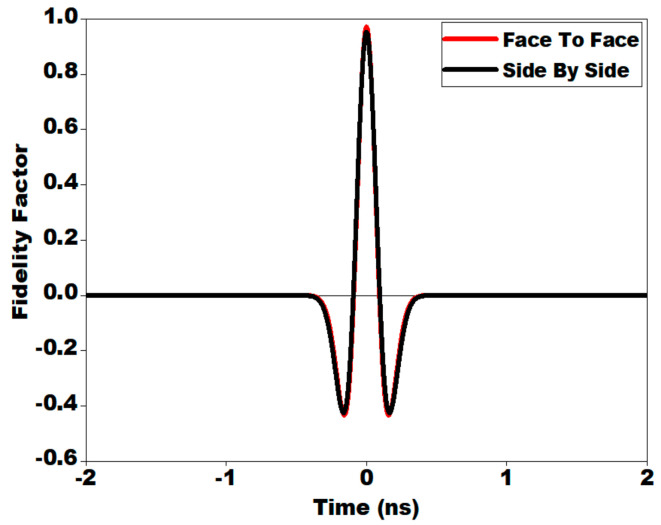
Variation of fidelity factor with regard to time for side by side and face to face arrangement.

Figure 32 presents the variation of FF with respect to time for both the configurations. From Figure 32, it is worth noting that the FF between transmitted and received pulses is around 97% and 95% in face to face and side by side arrangement, respectively. Higher value of the calculated fidelity factor ensures less distortion and good similarity between transmitted and received pulse.

### 6.1. Group Delay

**Figure 33:**
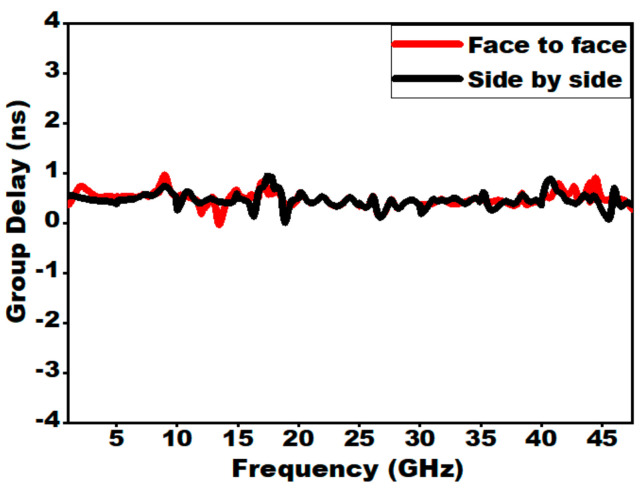
Group delay vs. frequency for the two configurations.

Group delay is another significant time domain parameter and it is computed mathematically as negative derivative of phase change with respect to frequency. As the signal transit through a device, it undergoes amplitude as well as phase distortion. A wave incident at the input of a device will have multiple frequency components. Thus, the group delay provides a degree of average time lag of input signal at each frequency. It also provides a measure of the dispersive nature of the device. Group delay also validates the linear phase response for the entire far-field region. For a good SWB system, the group delay should be as small as possible. Practically the group delay should be constant or its variation must be within 1 ns, which indicates a linear phase for the overall frequency range of operation. The linear phase response identifies the amount of distortion in the transmitted signal pulse. Group delay is calculated by using Equation (15).

(15) $$\tau_{g}(\omega )=-\frac{d\varphi (\omega )}{d\omega }=\frac{d\varphi (\omega )}{2\pi df}$$

where ϕ represents the transmitted signal phase response and ω denotes the frequency in radians per second. From Figure 33, it is clear that the presented antenna offers a group delay of less than 1 ns. The group delay is observed to be smooth for the designed antenna, which shows its excellent prevention capability against pulse dispersion.

### 6.2. Isolation

**Figure 34:**
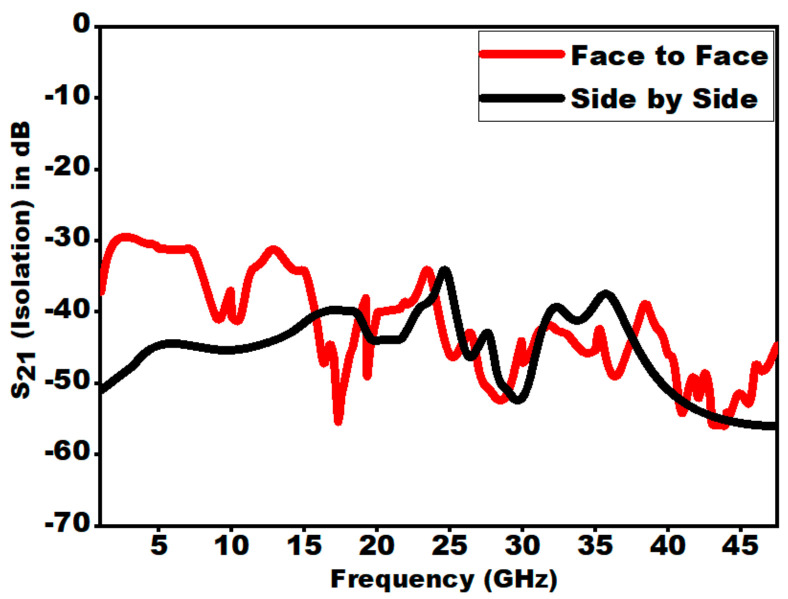
Isolation vs. frequency for the two configurations.

Isolation is a critical factor that signifies the coupling in the antenna structure. Figure 34 presents the isolation (S_21_) over the entire bandwidth for side by side and face to face configurations.

From Figure 34, it is seen that in both the configurations S_21_ < −30 dB. Hence, a large value of S_21_ indicates an unrelated transfer of signals at two ports in each of the arrangements. S_21_ of a two-port structure is calculated by using Formula (16).

(16) $$Isolation=-10\log_{10}{\left|S_{12}\right|}^{2}\;\mathrm{dB}\;$$

**Figure 35:**
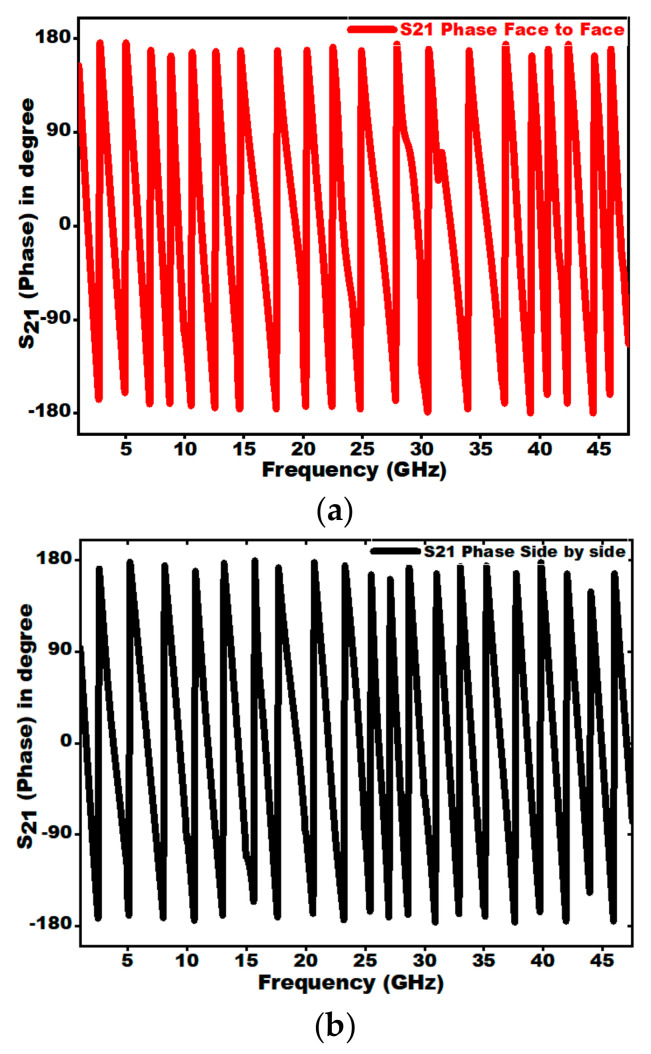
Phase of S_21_ variation in (**a**) face to face and (**b**) side to side configuration.

Figure 35a,b present the S_21_ phase variation for the two configurations. It can be clearly observed that the phase variation is linear for the overall range of frequency in both the configuration, which signifies that the pulse being received is without any out of phase components.

Table 5 incorporates and presents proposed antenna design comparison among other available SWB antenna relative to the electrical dimension, frequency range, fractional usable bandwidth, BDR, bandwidth ratio, peak gain and substrate material being utilized. According to Table 5, it is observed that the presented radiator has the maximum BDR in comparison with formerly reported SWB antenna.

## 7. Conclusions

A concentric Mickey-Mouse-structured monopole antenna is designed and investigated for wideband application. Parametric analysis has been conducted to extend the impedance bandwidth. A semi-elliptical ground plane along with notch is utilized to significantly improve the impedance bandwidth. The presented antenna structure achieves a BDR of 6596, signifying the highest BDR compared with all the formerly presented structures which validates its compact size and wider bandwidth. The proposed structure ensures simulated frequency range from 1.22 to 47.5 GHz with a fractional bandwidth of 190% and bandwidth ratio of 38.9:1. The measured frequency range is from 1.25 to 40 GHz with fractional bandwidth of 188% and BDR of 6523. Simulated gain varies from 0.5 to 10.3 dBi and measured gain varies from 0.2 to 9.7 dBi. The dimension, operating frequency, bandwidth percentage, bandwidth dimension ratio and peak realized gain of the presented antenna are compared with earlier existing designs. It is observed that the presented antenna has the optimized performance in comparison with other mentioned structures in the literature. By virtue of these advantages, the designed antenna finds its application in L, S, C, X, Ka, K, Ku, and Q band wireless communication systems. Further, the model of an equivalent circuit of the designed antenna is developed, and its corresponding S_11_ response has been investigated. The transient behavior of the designed antenna for pulsed operations has been characterized by time domain analysis which confirms the adequacy of the presented antenna for diverse wideband wireless application.

## Figures and Tables

**Table 1:** Optimal dimensions for different parameters of the designed antenna structure.

Symbol	Dimension (mm)	Symbol	Dimension (mm)
W	40	R4	2.6
L	45	R5	2.3
R1	9.7	Lf	21.4
R2	5.8	Wf	3.6
R3	4	Lg	15

**Table 2:** Outer ear position and segment lengths.

Outer Ear Center Position	Segment	Length (mm)
A1	A1-C	10.06
	A1-B1	4.5
	B1-C	9.0
A2	A2-C	11.18
	A2-B2	5.0
	B2-C	10.0
A3	A3-C	12.3
	A3-B3	5.5
	B3-C	11

**Table 3:** Inner ear position and segment lengths.

Inner Ear Center Position	Segment	Length (mm)
P1	P1-C	4.3
	P1-Q1	2.5
	Q1-C	3.5
P2	P2-C	5.0
	P2-Q2	3.0
	Q2-C	4.0
P3	P3-C	5.7
	P3-Q3	3.5
	Q3-C	4.5

**Table 4:** RLC component values for the antenna equivalent circuit.

Resonance. Frequency (GHz)	Z11 (Real) Ω	Z11 (Imaginary) Ω	L (pH)	C (pF)
3.4	51.117	2.3659	110.7	19.8
11.8	38.15	2.218	29.9	6.1
17.2	56.57	0.7732	7.2	12.0
20.2	41.16	1.218	9.6	6.5
24.8	61.31	3.336	21.4	1.9
30.3	59.54	1.6857	8.9	3.1
38.6	56.46	0.7444	3.1	5.5

**Table 5:** Comparison of the proposed super wideband radiator with different existing designs.

[Ref]	Size (λ Free Space Wavelength Calculated at Lower Frequency)	Freq. Range (GHz)	BW (%)	Bandwidth Ratio	BDR	Peak Gain (dBi)	Method of Antenna Design to Achieve SWB Operation	
							Radiator Design	Ground Plane Design
[3]	0.16 λ × 0.12 λ	0.4–20	192	50:1	10,000	6.3 at 17 GHz	3D volcano smoke shaped antenna	Symmetrical ground plane
[4]	0.4 λ × 0.25 λ	3–20	147	6.66:1	1470.0	6 at 6 GHz	Radiating patch with diamond-shaped slot	Rectangular slotted ground plane
[5]	1.28 λ × 4.9 λ	24–40	68	Not reported	3429.26	19 at 34GHz	Hybrid geometry radiator	Un-defected ground plane
[6]	0.24 λ × 0.32 λ	2.4–28.4	169	12:01	2200.5	7 at 18 GHz	Circular shaped patch radiator	Rectangular slotted ground plane
[8]	0.30 λ × 0.285 λ	2.25–11.05	132.33	4.9:1	1547.7	5.05 at 10 GHz	Polygonal-shaped patch radiator	Circular slotted ground plane
[9]	0.32 λ × 0.32 λ	0.64–16	184.6	25:1	1802.7	10 at 9 GHz	Half circular and half elliptical patch	Corner rounded ground plane
[10]	0.33 λ × 0.416 λ	2.5–110	191	44:1	1391.0	6 at 40 GHz	Hanning function-based tapered microstrip monopole antenna	Rectangular ground plane
[12]	0.18 λ × 0.22 λ	1.30–20	175.58	15.38:1	4261.0	5 at 20 GHz	Semicircular shaped radiator	Partial trapezoidal shaped ground plane
[13]	0.17 λ × 0.13 λ	0.96–10.9	167.22	11.35:1	6975.2	4.5 at 10 GHz	Partial circular monopole antenna with elliptical slot at the center	Notch loaded elliptical ground plane
[14]	0.38 λ × 0.55 λ	3–35	168	11.6:1	803.8	6 at 13 GHz	Propeller shaped monopole radiator	CPW-fed rectangular ground plane
[15]	0.326 λ × 0.265 λ	3.06–35	168	11.43	1944.6	Not reported	Inverted triangular-shaped patch antenna	Truncated rectangular ground plane with slits
[16]	0.32 λ × 0.34 λ	3.4–37.4	166.67	11:1	1531.8	11 at 32.5 GHz	Sierpinski hexagonal-shaped fractal antenna	CPW-fed ground plane
[18]	0.31 λ × 0.46 λ	3.15–32	164	10.16:1	1102.9	-3.2 at 5 GHz	Staircase-shaped patch antenna	CPW-fed modified ground plane
[19]	0.2785 λ × 0.234 λ	3.5–37.2	164	10:01	2541.1	13.7 at 33 GHz	Phi-shaped monopole patch antenna	Quarter elliptical CPW ground plane
[20]	0.256 λ × 0.247 λ	2.75–71	185	25.82:1	2912.2	12 at 60 GHz	Hexagonal-shaped radiator	CPW-fed asymmetric ground plane
[21]	0.17 λ × 0.13 λ	0.95–13.8	173.96	14.52:1	7871.4	6 at 13.8 GHz	Square monopole antenna which is modified by adding two stubs at the opposite side	CPW-fed corner truncated ground plane
[22]	0.35 λ × 0.23 λ	3–26	158	8.66:1	1962.7	7 at 26 GHz	Giusepe Peano fractal geometry radiator	Combination of circular and rectangular ground plane
[23]	0.23 λ × 0.25 λ	3.8–68	179	17.89:1	3015.0	13 at 48 GHz	CPW-fed octagonal-shaped fractal radiator	Defective ground structure
[24]	0.19 λ × 0.31 λ	3–60	181	20:1	3073.0	12 at 30 GHz	Circular-shaped metallic patch with Apollonius fractal geometry	Rectangular notch loaded ground plane
[25]	1.148 λ × 1.148 λ	17.22–180	165	10.45	125.2	10 at 110 GHz	Star snowflake structured fractal antenna	Rectangular ground plane
[26]	0.28 λ × 0.285 λ	1.4–20	173.8	14.2:1	2178.0	8 at 8.5 GHz	Equilateral triangle with inscribed circle fractal geometry radiator	Elliptical ground plane
[27]	0.33 λ × 0.36 λ	2–30	175	15:1	1473.0	Not reported	Octagonal shaped patch with koch fractal geometry	Rectangular ground plane
Prop.	0.16 λ × 0.18 λ	1.22–47.5	190	38.9:1	6596.5	10.3 at 40 GHz	Concentric-shaped radiating patch	Semi elliptical ground plane

## Data Availability

Not applicable.
